# APC/C^Cdh1^ is required for the termination of chromosomal passenger complex activity upon mitotic exit

**DOI:** 10.1242/jcs.251314

**Published:** 2020-09-15

**Authors:** Takaaki Tsunematsu, Rieko Arakaki, Hidehiko Kawai, Jan Ruppert, Koichi Tsuneyama, Naozumi Ishimaru, William C. Earnshaw, Michele Pagano, Yasusei Kudo

**Affiliations:** 1 Department of Oral Molecular Pathology, Tokushima University Graduate School of Biomedical Sciences, Tokushima 770-8504, Japan; 2 Department of Nucleic Acids Biochemistry, Hiroshima University Graduate School of Biomedical & Health Sciences, Hiroshima 734-8553, Japan; 3 Wellcome Centre for Cell Biology, Institute of Cell Biology, University of Edinburgh, Edinburgh EH9 3BF, Scotland, UK; 4 Department of Pathology and Laboratory Medicine, Tokushima University Graduate School of Biomedical Sciences, Tokushima 770-8504, Japan; 5 Department of Biochemistry and Molecular Pharmacology, New York University School of Medicine, New York, NY 10016, USA; 6 NYU Perlmutter Cancer Center, New York University School of Medicine, New York, NY 10016, USA; 7 Howard Hughes Medical Institute, New York University School of Medicine, New York, NY 10016, USA; 8 Department of Oral Bioscience, Tokushima University Graduate School of Biomedical Sciences, Tokushima 770-8504, Japan

**Keywords:** Chromosome passenger complex, Borealin, Aurora B, Ubiquitylation, APC/C^Cdh1^

## Abstract

During mitosis, the chromosomal passenger complex (CPC) ensures the faithful transmission of the genome. The CPC is composed of the enzymatic component Aurora B (AURKB) and the three regulatory and targeting components borealin, INCENP, and survivin (also known as BIRC5). Although the CPC is known to be involved in diverse mitotic events, it is still unclear how CPC function terminates after mitosis. Here we show that borealin is ubiquitylated by the anaphase promoting complex/cyclosome (APC/C) and its cofactor Cdh1 (also known as FZR1) and is subsequently degraded in G1 phase. Cdh1 binds to regions within the N terminus of borealin that act as a non-canonical degron. Aurora B has also been shown previously to be degraded by the APC/C^Cdh1^ from late mitosis to G1. Indeed, Cdh1 depletion sustains an Aurora B activity with stable levels of borealin and Aurora B throughout the cell cycle, and causes reduced efficiency of DNA replication after release from serum starvation. Notably, inhibition of Aurora B kinase activity improves the efficiency of DNA replication in Cdh1-depleted cells. We thus propose that APC/C^Cdh1^ terminates CPC activity upon mitotic exit and thereby contributes to proper control of DNA replication.

## Abstract (precis)

**Summary:** APC/C^Cdh1^ ubiquitylates both borealin, via a non-canonical degron motif, and Aurora B to terminate activity of the CPC in G1 phase. Disruption of this ubiquitylation reduces the efficiency of DNA replication.

## INTRODUCTION

The chromosomal passenger complex (CPC) plays an important role during mitosis to ensure the faithful segregation of genetic material into daughter cells (Earnshaw and Bernat, 1991). The CPC consists of four proteins: Aurora B (AURKB), INCENP (inner centromere protein), borealin (also known as CDCA8 and DASRA) and survivin (also known as BIRC5). The CPC proteins were originally defined by their pattern of localization to the chromosomes and spindle during mitosis. Among CPC proteins, Aurora B kinase is the enzymatic component, and the other three proteins are regulatory and targeting components (Vagnarelli and Earnshaw, 2004; Ruchaud et al., 2007).

Borealin was discovered in a proteomic screen for novel proteins associated with mitotic chromosome scaffolds (Gassmann et al., 2004) and, at the same time, in a screen for novel proteins capable of binding to chromosomes in *Xenopus* extracts (Sampath et al., 2004). The N terminus of human borealin participates in the three-­helix bundle that constitutes the CPC localization module (Jeyaprakash et al., 2007). Immunoprecipitation experiments reveal that survivin is associated with borealin in mitotic cells (Gassmann et al., 2004). Borealin also binds INCENP and might be involved in targeting the complex to centromeres. Borealin depletion by RNA interference increases the percentage of prometaphase cells (Bekier et al., 2015) and results in a dramatic increase in spindle–kinetochore misattachments and failures in cytokinesis (Gassmann et al., 2004; Sampath et al., 2004). These and a host of other observations indicate that the CPC regulates mitosis. However, it is still unclear how CPC activity is terminated after mitosis.

The APC/C (anaphase promoting complex/cyclosome) is a multi-subunit E3 ubiquitin ligase mainly active during mitosis and G1. It was originally identified as a ubiquitin ligase for cyclin B (King et al., 1995; Yu et al., 1996). Activity and substrate binding by the APC/C require the coactivator proteins, Cdc20 or Cdh1 (also known as FZR1) (Visintin et al., 1997). Cdc20 associates with the APC/C from prometaphase to anaphase and is responsible for the ubiquitylation of important mitotic regulators such as cyclin B, securin, and Kid (KIF22). Cdh1 maintains the activity of the APC/C from late anaphase through G1, targeting multiple substrates for degradation (Kramer et al., 1998). APC/C^Cdh1^ activity decreases at the onset of S phase, at which point inhibition by Emi1 (early mitotic inhibitor 1, also known as FBXO5) and other mechanisms prevent APC/C^Cdh1^ activity until the next late mitosis (Di Fiore and Pines, 2007; Machida and Dutta, 2007). Here, we show that borealin is ubiquitylated and targeted for degradation by APC/C^Cdh1^ during the G1 phase of the cell cycle.

## RESULTS

### Borealin protein is degraded via APC/C^Cdh1^ during G1

**Fig. 1.**
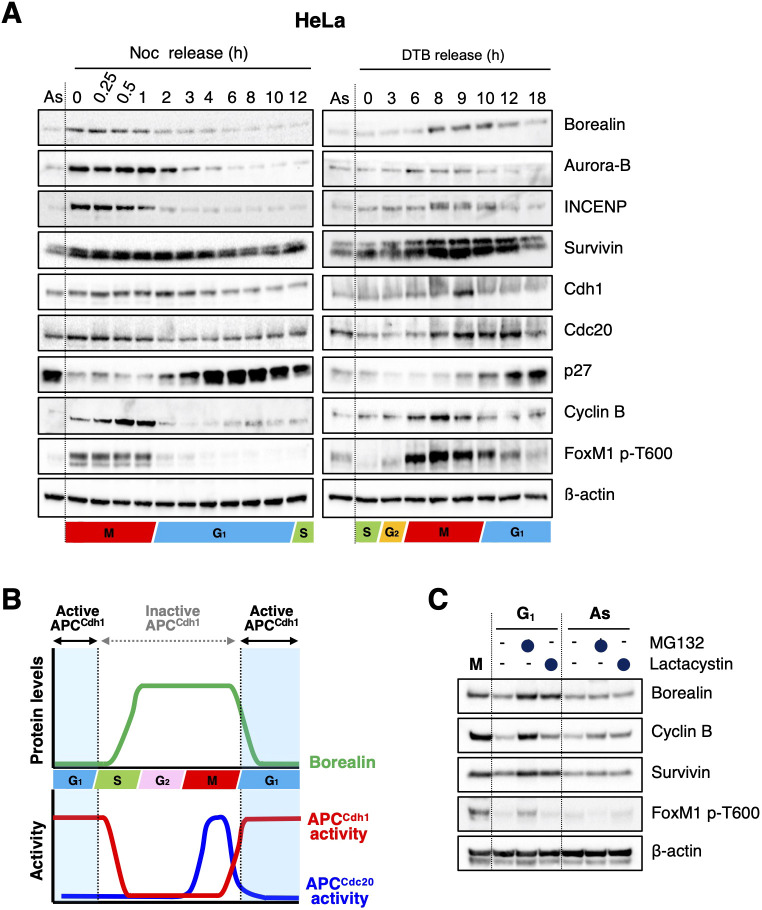
**Borealin is degraded at G1 phase via the ubiquitin–proteasome pathway.** (A) HeLa cells were released from a prometaphase arrest with nocodazole (Noc) and collected at the indicated times (left panel). In addition, HeLa cells were synchronized at the G1-S border using a double thymidine block (DTB). After release, cells were collected at the indicated time points (right panel). Cells were then lysed for immunoblotting as indicated. β-actin expression was used as a loading control. As; asynchronous. (B) The schematic graph shows protein expression level of borealin and APC/C activity during cell-cycle progression, based on the results shown in A. (C) HeLa cells were synchronized in M phase by mitotic shake-off with nocodazole (M). After 2 h release from mitotic arrest (G1), cells were treated with or without 10 μM MG132 or 10 μM lactacystin for 5 h. Asynchronous cells (As) were also treated with or without 10 μM MG132 or 10 μM lactacystin for 5 h. Cells were then collected and lysed for immunoblotting as indicated. β-actin expression was used as a loading control. Blots shown in A and C are representative of two experiments.

Borealin protein levels oscillate during the cell cycle; the protein accumulates during G2 and M phases and disappears in G1 (Fig. 1A,B). We examined the involvement of the ubiquitin–proteasome system (UPS) in the regulation of borealin protein levels during the cell cycle. Treatment of HeLa cells with either of two proteasome inhibitors (MG132 or lactacystin) resulted in borealin protein accumulation at 7 h after releasing HeLa cells from mitosis (Fig. 1C). This accumulation of borealin was not observed in asynchronous cells after treatment with MG132 or lactacystin (Fig. 1C).

**Fig. 2.**
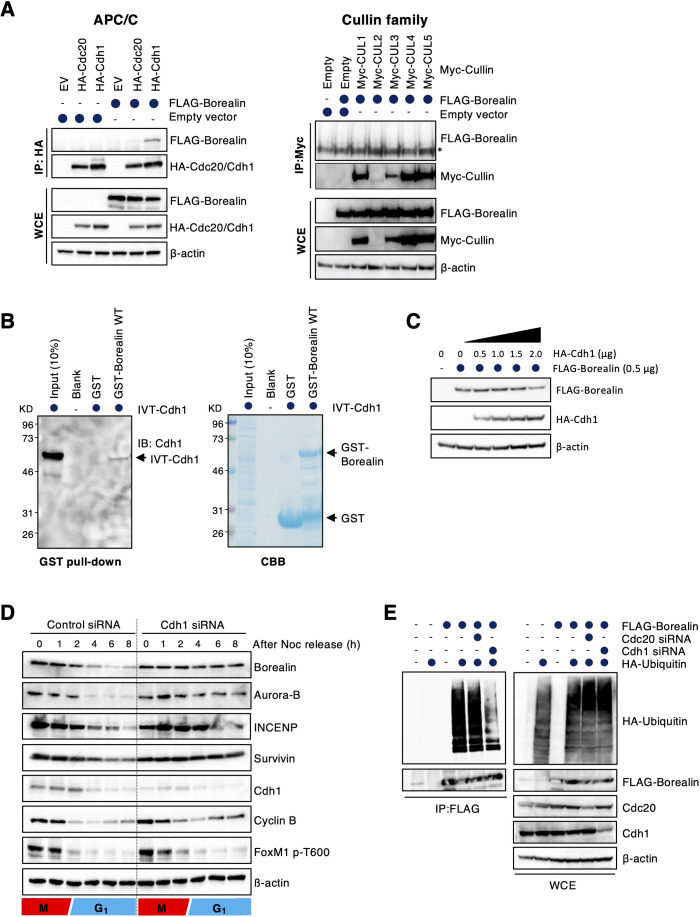
**Borealin is degraded at G1 phase via APC/C^Cdh1^-mediated polyubiquitylation.** (A) 293T cells were co-transfected with FLAG–Borealin and empty vector (EV), HA–Cdh1, HA–Cdc20, Myc–CUL1, Myc–CUL2, Myc–CUL3, Myc–CUL4, or Myc–CUL5. Then, cells were treated with 10 µM MG132 for 5 h. Cell extracts were immunoprecipitated (IP) with an affinity-purified polyclonal antibody against HA or Myc, and analyzed by immunoblotting as indicated. β-actin expression was used as a loading control. WCE: whole cell extracts. Asterisk indicates non-specific bands. (B) GST-tagged wild-type (WT) borealin bound to glutathione sepharose 4B resin was incubated with *in vitro* translated (IVT) Cdh1 for 2 h at 4°C. After washing, these proteins were eluted and analyzed by immunoblotting using anti-Cdh1 antibody. GST-tagged borealin and GST were visualized by Coomassie Blue (CBB) staining. IB, immunoblot. (C) HA-tagged Cdh1 (0, 0.5, 1.0, 1.5 and 2.0 μg) was co-transfected with FLAG-tagged borealin (0.5 μg) in 293T cells. Cells were then collected and lysed for immunoblotting as indicated. β-actin expression was used as a loading control. (D) HeLa cells were transfected with control or Cdh1 siRNA and synchronized at prometaphase arrest using nocodazole (Noc). After mitotic shake-off, cells were released and collected at the indicated times. Cell extracts were immunoblotted for the indicated proteins. β-actin expression was used as a loading control. (E) An *in vivo* ubiquitylation assay was performed. FLAG-tagged borealin and HA-tagged ubiquitin were co-transfected with either control, Cdc20 or Cdh1 siRNA in 293T cells. Cell extracts were immunoprecipitated using an anti-FLAG antibody, and the precipitates were blotted with anti-HA and anti-FLAG antibodies. Expression of endogenous Cdc20 and Cdh1 was also examined. β-actin expression was used as a loading control. Blots shown are representative of two experiments.

It is known that APC/C and multi-subunit cullin–RING ubiquitin ligase complexes are most intimately dedicated to basic cell-cycle control (Petroski and Deshaies, 2005). We first examined whether a specific cullin–RING complex or APC/C was involved in regulating borealin degradation. Borealin was found to co-immunoprecipitate with Cdh1 and an APC/C core subunit, Cdc27, but not Cdc20 (Fig. 2A; Fig. S1A). None of the cullin family proteins tested bound to borealin (Fig. 2A). Moreover, GST–borealin directly bound to *in vitro*-translated Cdh1 protein (Fig. 2B). The fact that borealin levels were found to be low in G1 and to rise during late S, G2 and M is consistent with the idea that borealin is an APC/C^Cdh1^ substrate (Fig. 1B). Indeed, APC/C^Cdh1^ substrates, including Aurora B and cyclin B, were also downregulated during G1 (Fig. 1A). Moreover, ectopic Cdh1 overexpression downregulated borealin levels (Fig. 2C; Fig. S1B), and Cdh1 depletion stabilized borealin protein during interphase (Fig. 2D). *In vivo* ubiquitylation of borealin was inhibited by Cdh1 knockdown, but not by Cdc20 knockdown (Fig. 2E). These findings indicate that borealin protein stability and levels are directly regulated by APC/C^Cdh1^.

### Two N-terminal regions in borealin are essential for Cdh1 binding and APC/C^Cdh1^-mediated polyubiquitylation

Next, we investigated the mechanistic details of the APC/C^Cdh1^-mediated degradation of borealin. Cdh1 activates the APC/C by contributing to substrate recognition (Hall et al., 2004; Zachariae, 2004). APC/C substrates contain one or more of several types of short peptide motifs with highly degenerate sequences that are required for their degradation, referred to as APC/C degrons. Borealin has three putative D-box (RxxL, where x indicates any amino acid) motifs, which could potentially be recognized by the APC/C^Cdh1^ (Fig. S2A). Wild-type borealin, individual D-box mutants and a mutant with all D-box motifs disrupted were all degraded following co-transfection of Cdh1 (Fig. S2B,C), suggesting that none of the three putative D-box motifs is responsible for the degradation of borealin protein.

**Fig. 3.**
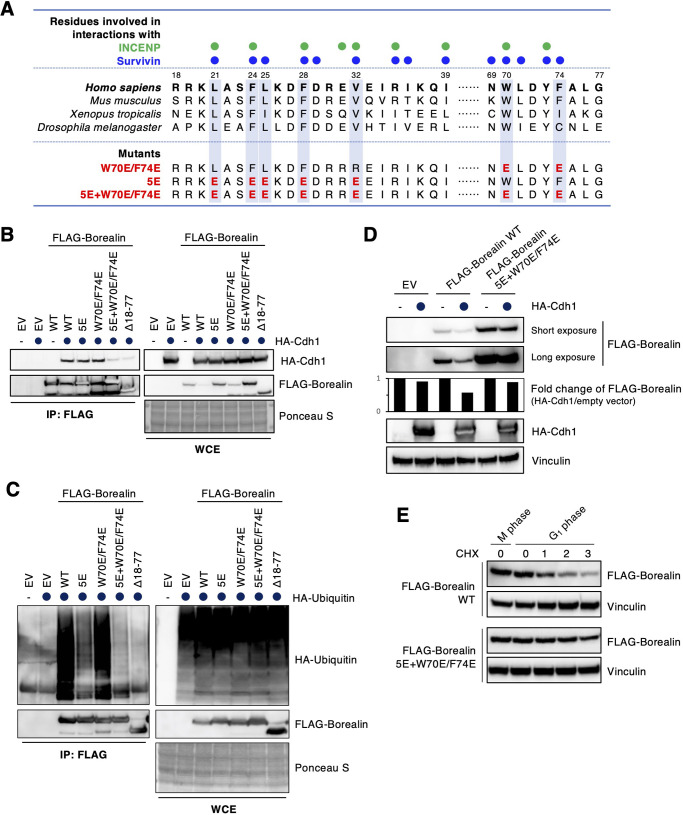
**N-terminal sequences of borealin are essential for Cdh1 binding and APC/C^Cdh1^-mediated polyubiquitylation.** (A) Comparison of borealin amino acid sequences of *Homo sapiens*, *Mus musculus*, *Xenopus tropicalis* and *Drosophila melanogaster* from residues 18 to 30 and 68 to 77 of the human sequence. Colored circles indicate residues involved in interactions with INCENP (green circles) and survivin (blue circles), as shown in a previous report (Jeyaprakash et al., 2007). (B) 293T cells were co-transfected with empty vector (EV), FLAG-tagged wild-type (WT) borealin, FLAG-tagged borealin 5E mutant (L21E/F24E/L25E/F28E/V32E), FLAG-tagged borealin W70E/F74E mutant, FLAG-tagged borealin 5E+W70E/F74E mutant (L21E/F24E/L25E/F28E/V32E/W70E/F74E), or FLAG-tagged borealin deletion mutant (Δ18–77) and HA–Cdh1 and treated with 10 μM MG132 for 5 h. Cell extracts were immunoprecipitated (IP) with an affinity-purified monoclonal antibody against FLAG and analyzed by immunoblotting as indicated. Ponceau S staining is shown as a loading control. WCE: whole cell extracts. (C) An *in vivo* ubiquitylation assay was performed. EV, FLAG-tagged WT borealin, or mutants were co-transfected with HA-tagged ubiquitin in 293T cells. Cell extracts were immunoprecipitated using an anti-FLAG antibody and analyzed by immunoblotting as indicated. Ponceau S staining is shown as a loading control. (D) EV, FLAG-tagged WT borealin, or the 5E+W70E/F74E borealin mutant were co-transfected with HA-tagged Cdh1 in 293T cells. Cell extracts were immunoblotted with anti-HA and anti-FLAG antibodies. Vinculin expression is shown as a loading control. Densitometric analysis of FLAG–borealin and vinculin was performed. The graph shows the fold change of FLAG–borealin:vincullin ratio in HA–Cdh1 transfected cells, compared with empty vector transfected cells. (E) HeLa cells transfected with FLAG-tagged WT borealin or FLAG-tagged borealin 5E+W70E/F74E mutant were synchronized at prometaphase using nocodazole (M phase). Cells were treated with 25 μg/ml cycloheximide (CHX) at 2 h after release from nocodazole block (G1 phase) and collected at indicated time points. Cell extracts were immunoblotted for the indicated proteins. Vinculin expression is shown as a loading control. Blots in B–E are representative of two experiments.

To find the borealin degron, we generated several deletion mutants (Fig. S3A) and evaluated the binding of these mutants to Cdh1 (Fig. S3B). This analysis revealed that the residues 18–39 and 69–77 were required for Cdh1 binding (Fig. S3A,B) and ubiquitylation (Fig. S3C). It has been shown that borealin residues W70 and F74 dock into the hydrophobic pocket present on the BIR (baculovirus inhibitor of apoptosis protein repeat) domain of survivin and have only marginal contacts with INCENP (Jeyaprakash et al., 2007). As previously reported (Jeyaprakash et al., 2007), mutants in which these residues were replaced by negatively charged amino acids (W70E/F74E) showed reduced binding to survivin (Fig. S4A,B). The W70E/F74E mutant was able to bind Cdh1, but the W70E/F74E mutant with an additional deletion of residues 18–39 (Δ18–39/W70E/F74E) could not bind to Cdh1, even though the Δ18–39 mutant did bind to Cdh1 (Fig. S4C). Thus, W70 and F74 contribute to Cdh1 binding. Because seven amino acids within residues 18–39 were found to be highly conserved among species, we generated 4A and 7A mutants (Fig. S4A). Both 4A and 7A mutants bound to Cdh1 (Fig. S4C). We then focused on all hydrophobic residues in this region (L21, F24, L25, F28 and V32), which are also highly conserved among species and are important for the interaction of borealin with survivin (Fig. 3A). By mutating these conserved hydrophobic residues to charged residues, the 5E mutant (L21E/F24E/L25E/F28E/V32E) and the 5E+W70E/F74E mutant (L21E/F24E/L25E/F28E/V32E with W70E/F74E) were generated (Fig. 3A). Binding of the 5E+W70E/F74E mutant to Cdh1 was substantially reduced, compared to binding of wild-type borealin, the W70E/F74E mutant and the 5E mutant (Fig. 3B). The borealin 5E+W70E/F74E mutant remarkably suppressed *in vivo* ubiquitylation (Fig. 3C). Computational analysis of the region comprising amino acids 20–31 of borealin, and its comparison with other APC/C^Cdh1^ degrons, suggested that borealin contains a non-canonical D box (Davey and Morgan, 2016) (Fig. S5).

Next, we examined whether the 5E+W70E/F74E mutant was degraded upon expression of ectopic Cdh1. Degradation of the 5E+W70E/F74E mutant was not promoted in Cdh1 co-transfection experiments (Fig. 3D). Notably, the expression level of the 5E+W70E/F74E mutant was higher than that of wild-type borealin despite transfecting the same amount of plasmid, suggesting that suppression of degradation might cause protein accumulation of the 5E+W70E/F74E mutant. To evaluate the stability of the 5E+W70E/F74E mutant, we examined its behavior during G1. Compared to wild-type borealin, the mutant was much more stable during G1 phase (Fig. 3E). We concluded that the 5E+W70E/F74E mutant is not marked for degradation via APC/C^Cdh1^.

### Survivin interferes with borealin ubiquitylation

**Fig. 4.**
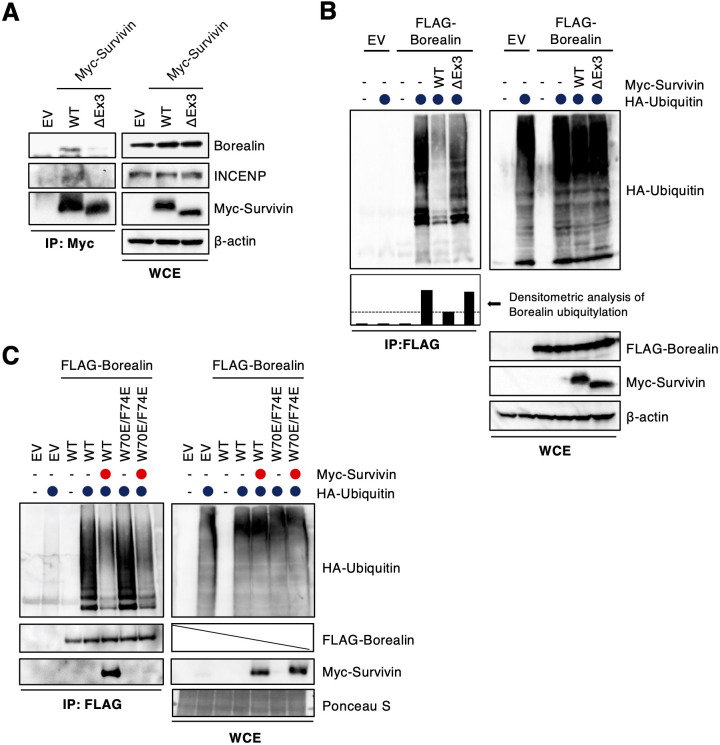
**Survivin interferes with borealin ubiquitylation.** (A) HeLa cells transfected with empty vector (EV), Myc-tagged wild-type (WT) survivin or the survivin ΔEx3 mutant were synchronized at prometaphase using nocodazole treatment. Cell extracts were immunoprecipitated (IP) using an anti-Myc antibody, and blotted with anti-borealin, anti-INCENP and anti-Myc antibodies. β-actin expression was used as a loading control. WCE, whole cell extracts. (B) An *in vivo* ubiquitylation assay was performed. FLAG-tagged WT borealin and HA-tagged ubiquitin were co-transfected with or without Myc-tagged WT survivin or survivin ΔEx3 mutant in 293T cells. Cell extracts were immunoprecipitated using an anti-FLAG antibody, and blotted with anti-HA, anti-FLAG and anti-Myc antibodies. β-actin expression was used as a loading control. The graph shows the densitometric analysis of borealin ubiquitylation. (C) An *in vivo* ubiquitylation assay was performed. FLAG-tagged WT borealin or the W70E/F74E borealin mutant and HA-tagged ubiquitin were co-transfected with or without Myc-tagged survivin in 293T cells. Cell extracts were immunoprecipitated using an anti-FLAG antibody, and blotted with anti-HA, anti-FLAG and anti-Myc antibodies. Ponceau S staining is shown as a loading control. Blots in A–C are representative of two experiments.

Based on the results of our co-immunoprecipitation experiments, two regions (amino acids 18–39 and 68–77) are important for Cdh1 binding. Interestingly, these regions are also essential for forming the core of the CPC together with survivin and INCENP (Jeyaprakash et al., 2007). In the globular part of the CPC core structure, borealin residues W70 and F74 dock into the hydrophobic pocket present on the BIR domain of survivin and have only marginal contacts with INCENP (Jeyaprakash et al., 2007). Indeed, wild-type survivin bound to borealin and INCENP (Fig. 4A). As previously shown (Jeyaprakash et al., 2007), the W70E/F74E mutant showed reduced binding with Aurora B, survivin, and INCENP, compared to the binding of wild-type borealin (Fig. S6A). Because L21, F24, F28 and V32 are residues involved in interactions with both INCENP and survivin (Fig. 3A), the 5E and 5E+W70E/F74E mutants, as expected, showed reduced binding with CPC components (Fig. S6A). As shown in Fig. 3C, ubiquitylation of the borealin 5E+W70E/F74E and Δ18–77 mutants was remarkably suppressed, when compared to that of the 5E and W70E/F74E mutants. These findings suggest that borealin ubiquitylation might depend on its interaction with survivin. Therefore, we examined the involvement of survivin in the ubiquitylation of borealin. Survivin depletion did not affect borealin ubiquitylation (Fig. S6B). Surprisingly, overexpressed wild-type survivin suppressed the ubiquitylation of borealin (Fig. 4B). Moreover, the splice variant ΔEx3 survivin, which does not bind to borealin (Noton et al., 2006), did not suppress the ubiquitylation of borealin (Fig. 4A,B), suggesting that Cdh1 might mainly recognize borealin in the complex with survivin, rather than free borealin. To prove this hypothesis, we examined whether the ubiquitylation of the borealin W70E/F74E mutant, which has reduced binding to survivin, was not suppressed by survivin overexpression. However, ubiquitylation of both the borealin W70E/F74E mutant and wild-type borealin was suppressed by survivin overexpression (Fig. 4C).

### Sustained CPC activity induces early DNA replication

**Fig. 5.**
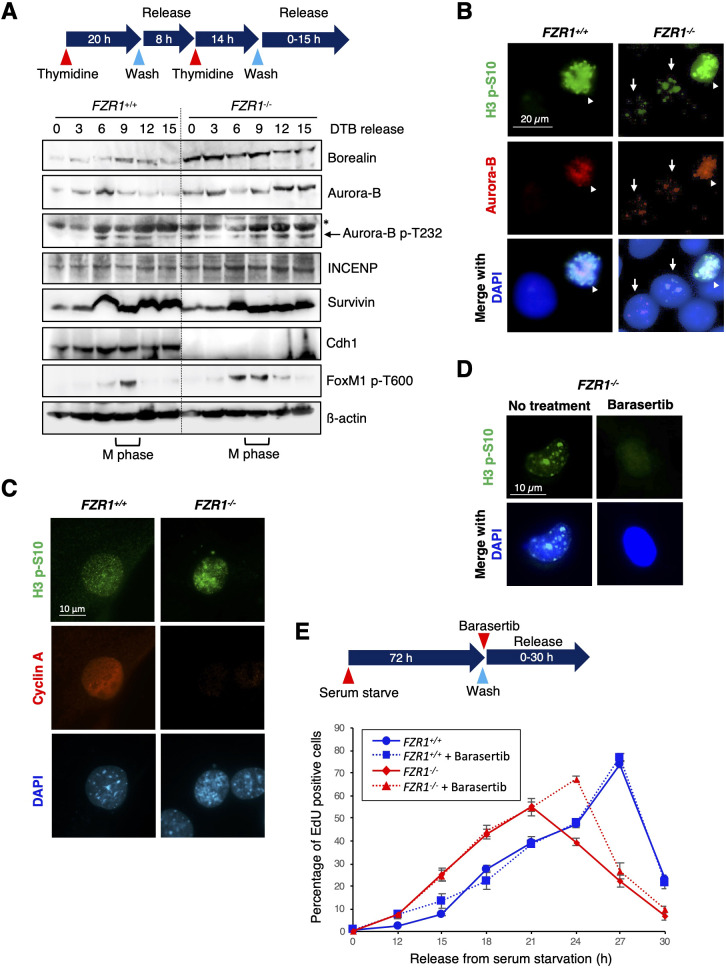
**Cdh1 depletion induces early DNA replication.** (A) *Fzr1*^+/+^ and *Fzr1*^−/−^ MEFs were synchronized at G1-S phase by double thymidine block (DTB, see diagram). After release from G1-S arrest, cells were collected at the indicated times. Cell extracts were immunoblotted for the indicated proteins. M phase is evaluated by detection of FoxM1 p-T600. β-actin is shown as a loading control. Asterisk shows the position of Aurora A p-T288. (B) Subcellular colocalization of Aurora B and histone H3 p-S10 in *Fzr1*^+/+^ and *Fzr1*^−/−^ MEFs. Cells were fixed and stained using anti-H3 p-S10 (green) and anti-Aurora B (red) antibodies. Nuclei of the cells were stained using DAPI (blue). Arrowheads show H3 p-S10 in mitotic cells, and arrows show H3 p-S10 in interphase cells. Scale bar: 20 µm. (C) *Fzr1*^+/+^ and *Fzr1*^−/−^ MEFs were fixed and stained using anti-H3 p-S10 (green) and anti-cyclin A (red) antibodies. Nuclei of the cells were stained with DAPI (blue). Cyclin A expression was used as a marker of G2 cells. Scale bar: 10 µm. (D) *Fzr1*^−/−^ MEFs were treated with the Aurora B specific inhibitor barasertib (10 nM) for 24 h. Cells were fixed and stained using an anti-H3 p-S10 antibody (green). Nuclei of the cells were stained using DAPI (blue). Scale bar: 10 µm. (E) *Fzr1*^+/+^ and *Fzr1*^−/−^ MEFs were serum starved for 72 h, forced to enter the cell cycle after stimulation with 15% FBS and pulsed with EdU. The percentage of EdU-positive cells was scored at different time points. Three independent experiments were used to calculate the mean±s.d. Blots and images in A–D are representative of at least two experiments.

Previous reports show that Aurora B is also degraded by the APC/C^Cdh1^ from late mitosis to G1 (Nguyen et al., 2005; Stewart and Fang, 2005; Floyd et al., 2013). Indeed, Cdh1 depletion stabilized Aurora B protein during interphase (Fig. 2D). Present and previous findings indicate that APC/C^Cdh1^ regulates the protein level of borealin and Aurora B after mitosis. However, it is still unclear how CPC function terminates after mitosis. To determine the involvement of APC/C^Cdh1^ in the termination of CPC function, we used *Fzr1* (which encodes Cdh1) knockout mouse embryonic fibroblasts (MEFs). First, we examined whether borealin and Aurora B were stabilized in interphase of *Fzr1*^−/−^ MEFs. As expected, levels of borealin and Aurora B oscillated in *Fzr1*^+/+^ MEFs but were more stable throughout the cell cycle in *Fzr1*^−/−^ MEFs (Fig. 5A). Because CPC functions are dependent on the action of Aurora B kinase (Vagnarelli and Earnshaw, 2004; Ruchaud et al., 2007), we examined Aurora B activity by detecting its activating phosphorylation on T232 (p-T232). In *Fzr1*^+/+^ MEFs, Aurora B was phosphorylated mostly around mitosis. In contrast, in *Fzr1*^−/−^ MEFs, Aurora B p-T232 was detected in interphase (Fig. 5A). It is well known that the mitotic phosphorylation of histone H3 at S10 (H3 p-S10) is governed by Aurora B (Crosio et al., 2002). To asses CPC activity throughout the cell cycle, we examined the levels of H3 p-S10 in G1, where H3 p-S10 is not observed. Large numbers of interphase nuclear foci of H3 p-S10 and Aurora B were observed in *Fzr1*^−/−^ MEFs, compared to *Fzr1*^+/+^ MEFs (Fig. 5B). It has recently been shown that nuclear foci of H3 p-S10 become visible in late S phase and G2 (Ruppert et al., 2018). Indeed, nuclear foci of H3 p-S10 were observed in the cells in G2 (cells with cyclin A expression) (Fig. 5C). However, in *Fzr1*^−/−^ MEFs, nuclear foci of H3 p-S10 were also observed in the cells in G1 (cells without cyclin A expression) (Fig. 5C). These nuclear foci of H3 p-S10 disappeared upon treatment with the Aurora B kinase inhibitor, barasertib (Fig. 5D), suggesting that APC/C^Cdh1^ is required for termination of CPC activity.

It has previously been shown that DNA replication is less efficient in Cdh1 (*Fzr1*)*-*deficient MEFs (García-Higuera et al., 2008). To determine the involvement of CPC activity in this phenotype of *Fzr1*^−/−^ MEFs, we examined whether this phenotype could be rescued by barasertib treatment. DNA replication was assessed by measuring EdU nucleotide incorporation after serum starvation of *Fzr1*^−/−^ and *Fzr1*^+/+^ MEFs. After re-adding serum, *Fzr1*^−/−^ MEFs entered S phase with kinetics different to those of *Fzr1*^+/+^ MEFs. As previously reported (García-Higuera et al., 2008), a greater number of *Fzr1*^−/−^ MEFs than *Fzr1*^+/+^ MEFs had entered S phase 21 h after serum stimulation (55% versus 40%) (Fig. 5E). Moreover, the entry of *Fzr1*^−/−^ MEFs into S phase peaked at 21 h (55% of cells), compared with a peak at 27 h for *Fzr1*^+/+^ MEFs (74% of cells) (Fig. 5E). Thus, DNA replication was apparently less efficient in *Fzr1*^−/−^ MEFs, compared with *Fzr1*^+/+^ MEFs. Interestingly, barasertib treatment shifted the peak of S phase in *Fzr1*^−/−^ MEFs from 21 h to 24 h and improved the efficiency of DNA replication from 55% to 68% (Fig. 5E). These findings suggest that sustained CPC activity after mitosis might reduce the efficiency of DNA replication.

## DISCUSSION

Here, we have shown that borealin protein stability and levels are directly regulated by APC/C^Cdh1^. APC/C substrates contain one or more of several types of short peptide motifs with highly degenerate sequences, referred to as APC/C degrons. Borealin ubiquitylation does not require a canonical D-box motif and/or a functional KEN box, which are recognized by Cdh1. It appears that seven hydrophobic residues (L21, F24, L25, F28, V32, W70, and F74) are essential for Cdh1 binding. Computational analysis of the region comprising amino acids 20–31 of borealin (^20^KLASFLKDFDRE^31^) and its comparison with other APC/C^Cdh1^ degrons suggested that borealin contains a non-canonical D box (Fig. S5). Among APC/C^Cdh1^ degrons, a leucine residue within the canonical D-box sequence (RxxL) may be common. Indeed, a single-alanine-substitution of L295 within the O-box (^291^PASPLTEKNAK^301^) in *Drosophila* ORC1 and L26 within the meiotic regulator Spo13 degron sequence (^26^LxExxxN^32^) eliminate their polyubiquitylation (Araki et al., 2005; Sullivan and Morgan, 2007). For borealin ubiquitylation, at least four hydrophobic residues within the non-canonical D box (^20^KLASFLKDFDRE^31^) are involved (Fig. S5).

Previous reports show that human cyclin A and yeast Clb5 each contain a D box, an ABBA motif and a potential degenerate KEN box, but also employ the Cdk-associated subunit Cks1 (CSK1B in humans) to provide additional binding affinity for the APC/C (Lu et al., 2014; Di Fiore et al., 2015). Therefore, borealin might require residues W70 and F74, which are essential for the interaction with survivin, for additional binding affinity to Cdh1. However, survivin does not act as an adaptor of borealin ubiquitylation (Fig. S6B). Surprisingly, wild-type survivin, but not the ΔEx3 mutant, suppressed borealin ubiquitylation (Fig. 4B), suggesting that the inhibitory effect on borealin ubiquitylation might be dependent on the physical interaction between borealin and survivin. However, survivin overexpression suppressed ubiquitylation of the borealin W70E/F74E mutant, which shows less binding with survivin, as well as wild-type borealin (Fig. 4C). Therefore, we suggest that survivin may suppress the borealin ubiquitylation via interaction with Cdh1, but not via direct interaction with borealin. To prove this hypothesis, further experiments will be required.

The protein levels of Aurora B and borealin are regulated by the UPS. During mitosis, Aurora B is ubiquitylated by two midzone-associated E3 ubiquitin ligase complexes, CUL3–KLHL9–KLHL13 (Sumara et al., 2007) and CUL3–KLHL21 (Maerki et al., 2009). Ubiquitylated Aurora B is subsequently removed from anaphase chromosomes by the AAA+ ATPase Cdc48 (cell division control protein 48; also known as p97 and VCP) and its adaptor proteins Ufd1–Npl4 (Ramadan et al., 2007; Dobrynin et al., 2011). This process is thought to contribute to determining the levels and distribution of the CPC on chromosomes before mitotic exit. From late mitosis to G1, Aurora B is ubiquitylated by APC/C^Cdh1^ via intact KEN-box and A-box motifs (Nguyen et al., 2005; Stewart and Fang, 2005). These findings indicate that distinct ubiquitin ligases regulate different pools of Aurora B in space and time for orchestration of proper control of mitosis. Surprisingly, formation of the CPC is not always necessary for Aurora-B activity (Mackay et al., 2010). Upon mitotic exit, APC/C^Cdh1^-mediated degradation of borealin and Aurora B might trigger the termination of CPC function and Aurora B activity.

Faithful chromosome segregation during cell division is crucial for growth, development and reproduction. Aneuploidy arising from chromosomal instability (CIN) has been assumed to contribute to carcinogenesis. Indeed, animal models have shown that failure to control either chromosome segregation or cytokinesis can cause CIN and cell transformation (Fujiwara et al., 2005; Iwanaga et al., 2007; Li et al., 2009; Weaver et al., 2007). Aurora B is overexpressed in various types of cancer, resulting in multi-nucleation and polyploidy (Portella et al., 2011; Nguyen et al., 2009). Also, borealin is overexpressed in colon and gastric cancers (Chang et al., 2006; Wang et al., 2014). Therefore, degradation of borealin and Aurora B, promoted by the APC/C^Cdh1^ after mitosis, and subsequent timely termination of CPC activity might be important for preventing oncogenic events. Indeed, Cdh1-heterozygous animals have increased susceptibility to spontaneous tumors (García-Higuera et al., 2008). Moreover, Cdh1-deficient cells show early onset of DNA replication and replication stress (García-Higuera et al., 2008; Sigl et al., 2009). Interestingly, the abnormal DNA replication in *Fzr1*^−/−^ MEFs was rescued by Aurora B kinase inhibitor treatment (Fig. 5B), suggesting that termination of CPC activity might contribute to proper control of DNA replication.

**Fig. 6.**
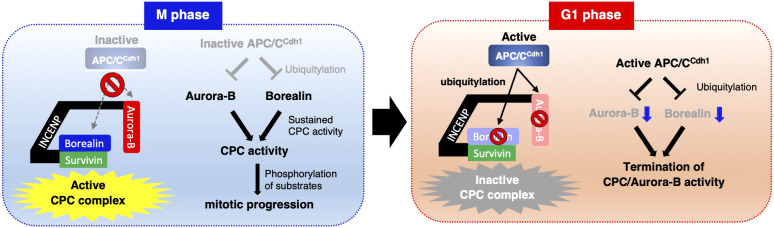
**Schematic model for CPC regulation from M phase to G1 phase.** In M phase, APC/C^Cdh1^ is inactive. Because Aurora B and borealin are stable, CPC is active. In G1 phase, borealin and Aurora B are ubiquitylated by APC/C^Cdh1^, and CPC activity is terminated. The termination of CPC function via degradation of borealin and Aurora B by APC/C^Cdh1^ might be required for proper cell cycle control.

In conclusion, we demonstrated that fluctuating borealin protein levels across the cell cycle are regulated by APC/C^Cdh1^. Cdh1 recognizes a unique non-canonical D-box sequence in borealin. Because Aurora B protein levels are also regulated by APC/C^Cdh1^ from late mitosis to G1, APC/C^Cdh1^ might play an important role for termination of CPC function and Aurora B activity upon mitotic exit (Fig. 6). Whether abnormal accumulation of borealin and/or Aurora B due to defects in APC/C^Cdh1^ regulation contributes to cancer etiology requires further studies.

## MATERIALS AND METHODS

### Cell culture and synchronization

293T and HeLa cells were obtained from the American type culture collection (ATCC). U2OS cells conditionally expressing Myc–Cdh1 were provided by Dr Jiri Lukas (Institute for Cancer Biology and Center for Genotoxic Stress, Danish Cancer Society, Copenhagen, Denmark). Wild-type and *Fzr1*^−/−^ MEFs obtained from conditional knockout mice with a targeted mutation in the *Fzr1* locus were provided by Dr Marcos Malumbres (National Cancer Research Centre, Madrid, Spain) (García-Higuera et al., 2008). 293T, HeLa, U2OS cells and MEFs were maintained in Dulbecco's Modified Eagle's Medium (D-MEM) (WAKO, Japan) supplemented with heat-inactivated 10% fetal bovine serum (FBS) (Invitrogen, San Diego, CA), at 37°C in 5% CO_2_.

For prometaphase arrest, HeLa cells were synchronized at prometaphase using 50 ng/ml nocodazole (Sigma-Aldrich) treatment for 12 h followed by mitotic shake-off. Subsequently, synchronized cells were released from the mitotic arrest by washing out nocodazole. Cells were collected at the indicated time points. HeLa cells were synchronized at the G1-S border using a double thymidine block. In brief, cells were incubated for 20 h in 2 mM thymidine (Sigma-Aldrich), released for 8 h, and then incubated for another 14 h in 2 mM thymidine. Cells were released from G1-S arrest by washing out thymidine. At 6 h following release, cells were treated with 50 ng/ml nocodazole for 12 h followed by mitotic shake-off. Subsequently, synchronized cells were released from the mitotic arrest by washing out nocodazole. Cells were collected at the indicated time points.

MEFs were synchronized at the G1-S border using a double thymidine block. In brief, cells were incubated for 20 h in 2 mM thymidine (Sigma-Aldrich), released for 8 h, and then incubated for another 14 h in 2 mM thymidine. Cells were release from G1-S arrest by washing out thymidine. Cells were collected at the indicated time points.

### Reagents and antibodies

MG132 (Z-Leu-Leu-Leu-CHO) and lactacystin were purchased from Peptide Institute Inc. (Osaka, Japan). Cycloheximide (CHX) was purchased from Sigma. Doxycycline was purchased from Clontech. Aurora B-specific inhibitor, barasertib (AZD1152-HQPA) was obtained from Selleckchem. For detection of borealin, an anti-borealin antiserum raised in rabbit against human full-length borealin protein was previously generated (Gassmann et al., 2004). Commercial antibodies used were: mouse monoclonal anti-Aurora A, anti-Aurora B, and anti-p27^Kip1^ antibodies (#610938, #611082 and #610242, respectively; BD Transduction Laboratories); rabbit polyclonal anti-INCENP (#2786; Cell Signaling Technology); rabbit monoclonal anti-FoxM1 p-T600, anti-HA, anti-Aurora A p-T288/Aurora B p-T232/Aurora C p-T198, and anti-vinculin antibodies (#14655, #3724, #2914 and #13901, respectively; Cell Signaling Technology); anti-Cdc23 and anti-INCENP antibodies (#ab72206 and #ab12183; Abcam); rabbit monoclonal anti-Aurora B (#ab45145; Abcam); mouse monoclonal anti-Cdh1/Fzr and anti-Cdc20 antibodies (#K0085-3 and #K0140-3, respectively; MBL); rabbit polyclonal anti-cyclin A antibody (#sc-751; Santa-Cruz Biotechnology); mouse monoclonal anti-cyclin B1 antibody (#sc-245; Santa-Cruz Biotechnology); anti-Cdc27 and anti-β-actin antibodies (#C7104 and #A5441, respectively; Sigma-Aldrich); mouse monoclonal anti-HA, anti-FLAG, anti-c-Myc, and anti-GFP antibodies (#014-21881, #018-22381, #011-21874 and #012-22541, respectively; Wako); rabbit polyclonal anti-survivin antibody (#NB500-201; Novus Biologicals); rabbit polyclonal anti-Cul1 antibody (#71-8700; Thermo Fisher Scientific); and rabbit polyclonal anti-H3 p-S10 antibody (#06-570; Merck Millipore).

### Plasmids

pEGFP-N1 plasmid encoding full-length human borealin was used for constructing three different borealin D-box mutants; D1 (RRKL>ARKA), D2 (RQNL>AQNA) and D3 (RKNL>AKNA) using the KOD plus mutagenesis kit (Toyobo, Osaka, Japan). The pEGFP-N1-borealin plasmid was digested by *Xho*I and *Kpn*I followed by ligation with *Xho*I and *Kpn*I-digested pcDNA3.1 vector (Invitrogen) resulting in pcDNA3.1-borealin plasmid. A FLAG-tag sequence was then inserted at the C terminus of borealin using the KOD plus mutagenesis kit (Gassmann et al., 2004). For generating various borealin mutants, pcDNA3.1-FLAG-tagged-borealin was mutated using the KOD plus mutagenesis kit. For production of borealin recombinant protein, borealin cDNA was inserted to the EcoR1 site of the pGEX-4T-1 vector (GE Healthcare). pcDNA3-HA-tagged human ubiquitin was kindly provided by Dr Anindya Dutta (University of Virginia, Charlottesville, VA). pCMV-HA-tagged Cdh1 and Cdc20 were kindly provided by Dr Kristian Helin (Memorial Sloan Kettering Cancer Institute, New York, NY). For *in vitro* translation, Cdh1 cDNA was cloned into the pcDNA3.1 vector. pcDNA4-Myc tagged wild-type and ΔEx3 survivin were provided by Dr Rachel Altura (The Ohio State University, Columbus, OH).

### DNA and siRNA transfection

DNA transfection or co-transfection into mammalian cells was conducted using XtremeGENE HP transfection reagent (Roche) or PEI-Max (Polysciences, Inc), according to the manufacturer's instructions. 30 nM of siRNA were transfected using Oligofectamine^®^ RNAi MAX (Invitrogen), according to the manufacturer's instructions. Cdh1 and Cdc20 siRNAs were obtained from Dharmacon as the following sequences: Cdh1, 5′-UCUGGUGGACUGGUCGUCC-3′; Cdc20, 5′-GCCGGCAGGACUCCGGGCCGA-3′. Survivin siRNA was obtained from Ambion as the following sequence: 5′-CAAAGGAAACCAACAAUAA-3′. The negative control (siTrio negative control siRNA) was obtained from Cosmo Bio Inc. (Tokyo, Japan).

### Immunoprecipitation and immunoblot analysis

Cells were lysed using CPC lysis buffer (50 mM Tris-HCl pH 7.6, 400 mM NaCl, 0.5% Nonidet P-40, 10 mM NaF, 0.1% sodium deoxycholate, 1.5 mM MgCl_2_, 100 nM okadic acid and 40 mM β-glycerophosphate) with protease inhibitors and incubated on ice for 30 min adding Turbo nuclease (Accelagen). After incubation, cells were centrifuged for 20 min at 13,200 rpm (17,000 ***g***) and the supernatant was used as cell lysate.

For immunoprecipitation, cell lysate was diluted in NP-40 lysis buffer (10 mM Tris-HCl pH 7.6, 150 mM NaCl, 0.5 mM EDTA, 10 mM NaF and 0.1% Nonidet P-40) with protease inhibitors and immunoprecipitated using the specific antibody against the target protein. The immunoprecipitates were subjected to immunoblot analysis. For immunoblotting, 30 µg of protein was subjected to 10–20% gradient polyacrylamide gel (ATTO) electrophoresis followed by electroblotting onto a nitrocellulose membrane (GE Healthcare). To detect the immunocomplex, an Immobilon HRP substrate (Merck Millipore) or ImmunoStar LD (Wako) was used. Primary antibodies for immunoblot analysis were used at dilutions of 1:500 for Cdh1 and Cdc20, 1:1000 for borealin, INCENP, p27, Cyclin B, FoxM1 p-T600, and Aurora-B p-T232, 1:2000 for Aurora-B, survivin, vinculin, FLAG, HA, and Myc, and 1:10,000 for β-actin. Primary antibodies were used for immunoprecipitation analysis at dilutions of 1:250 for HA and FLAG, and 1:500 for Myc.

### GST pulldown assay

To prepare *in vitro* translated (IVT) Cdh1 recombinant protein, the TNT Coupled Wheat Germ Extract System (Promega) was used with pcDNA3.1-Cdh1 vector, following the manufacturer's protocol. For GST-tagged recombinant protein production, pGEX-4T-1-borealin vector was transformed to BL21(DE3) (Nippon gene) and GST-tagged borealin was expressed for 15 h at 18°C with 0.2 mM isopropyl-1-thio-D-galactopyranoside (IPTG). Cells were lysed using NETN buffer (100 mM NaCl, 1 mM EDTA, 50 mM Tris-HCl pH 7.5, 1 mM PMSF, 5 mM benzamidine and 0.5% NP-40) and sonicated. After centrifugation, cleared lysate was collected and incubated in GST SpinTrap columns (GE healthcare) for 1 h at 4°C. Then, each column was washed three times with binding buffer (10 mM Tris-HCl pH 7.6, 150 mM NaCl, 0.5 mM EDTA, 10 mM NaF, 0.1% Nonidet P-40, 20 mM β-glycerophosphate and 20 nM okadic acid) and incubated with 15 μl IVT Cdh1 and binding buffer for 2 h at 4°C. After incubation, each column was washed five times with binding buffer and proteins were eluted using GST elution buffer (GE healthcare). The eluted proteins were analyzed using SDS–PAGE.

### *In vivo* ubiquitylation assay

293T cells transfected with FLAG–borealin and HA–ubiquitin were treated with 40 μM MG132 for 1 h at 36 h following transfection. Cells were lysed with denaturing ubiquitination buffer (50 mM Tris-HCl pH 7.6, 5 mM DTT and 1% SDS) and immediately boiled for 10 min at 95°C. After denaturing, cell lysates were sonicated and centrifuged for 20 min at 13,200 rpm (17,000 ***g***). The supernatant was diluted using immunoprecipitation buffer (50 mM Tris-HCl pH 7.6, 5% glycerol, 150 mM KCl and 0.4% Nonidet P-40) with protease inhibitors, then immunoprecipitated using anti-FLAG monoclonal antibody (Wako). Immunoprecipitated ubiquitylated proteins were fractionated using SDS–PAGE, transferred, and immunoblotted using anti-HA antibody.

### Immunofluorescence

Cells were grown on glass coverslips, fixed in methanol:acetone (1:1) for 10 min. After blocking using 3% BSA, the coverslips were incubated with primary antibodies. Primary antibody dilutions used were 1:100 for Aurora-B, and 1:200 for H3 p-S10 and Cyclin A. Alexa Fluor 488-conjugated anti-rabbit IgG and Alexa Fluor 555-conjugated anti-mouse IgG, or Alexa Fluor 488-conjugated anti-mouse IgG and Alexa Fluor 555-conjugated anti-rabbit IgG were used as secondary antibodies (Invitrogen). DNA was visualized using 4′,6-diamidino-2-phenylindole (DAPI) staining. Immunostaining of the cell preparations was recorded using an epifluorescence Zeiss Axioplan 2 microscope (Zeiss, Inc., Thornwood, NY), with Plan-Neofluar 40×/0.75 Ph2 and 63x Plan-Apochromat 63×/1.4 NA oil-immersion objective lenses, attached to a charge-coupled-device camera.

### EdU staining

For EdU incorporation experiments, MEF cells were synchronized in G0 phase by serum starvation. After 72 h, cells were treated with 15% FBS and pulsed with EdU. EdU staining was performed using a Click-iT Plus EdU Flow Cytometry Assay Kit (Life Technologies) according to the manufacturer's protocol. Briefly, cells were treated with 10 µM EdU for 30 min before each indicated time point. MEFs were then fixed in 4% paraformaldehyde for 15 min, stained with Alexa Fluor 488–picolyl azide and analyzed by flow cytometry. Three independent experiments were used to calculate mean±s.d.

## Supplementary Material

Supplementary information

Reviewer comments
