# Supplementary material for: APC/CCdh1 is required for the termination of chromosomal passenger complex activity upon mitotic exit
Source: J Cell Sci. 2020 Sep 15;133(18):jcs251314. doi: 10.1242/jcs.251314 (PMC7520452; doi:10.1242/jcs.251314)
Supplement: Supplementary information [file joces-133-251314-s1.pdf]

Figure S1

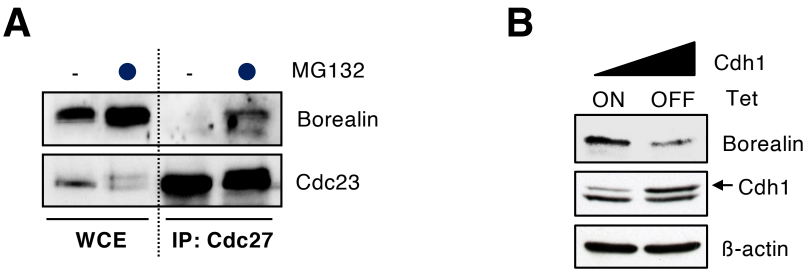

Figure S1. Borealin is degraded by APC/C^{Cdh1}. (A) HeLa cells were synchronized by mitotic shake-off with nocodazole. After 2 hours released from mitotic arrest, cells were treated with or without 10 µM of MG132 for 5 hours. Cell extracts were immunoprecipitated with anti-Cdc27 antibody, and precipitants were blotted with anti-Borealin and anti-Cdc23 antibodies. (B) U2OS-tet-Cdh1 cells were collected before and after the removal of tetracycline (Tet). The protein levels of Borealin and Cdh1 were examined by immunoblotting. ß-actin expression was used as a loading control.

Journal of Cell Science • Supplementary information

Figure S2

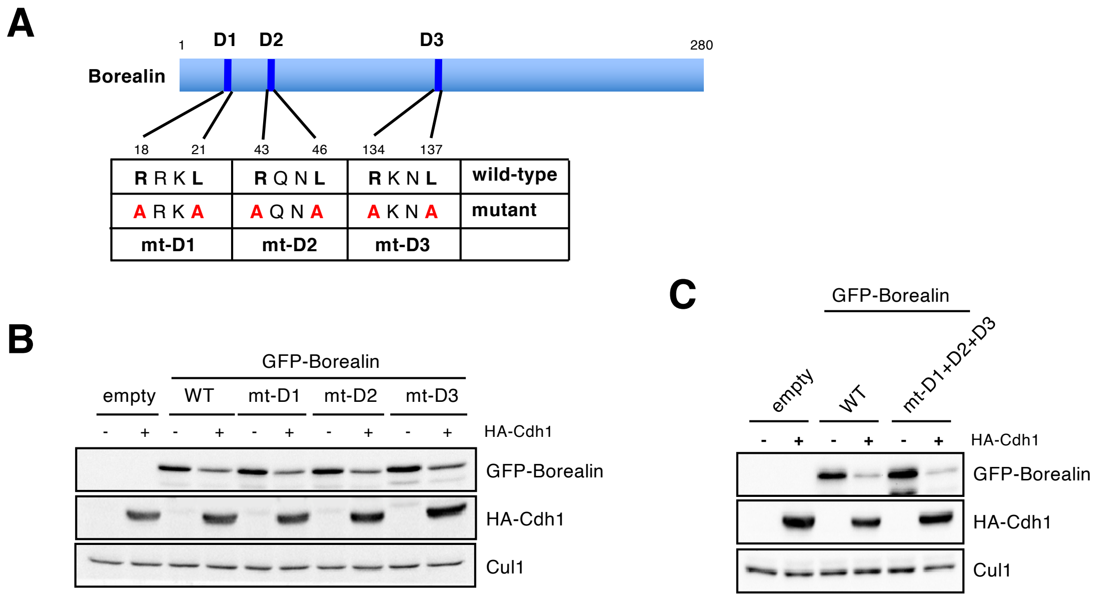

Figure S2. Three putative D-Box motifs of Borealin cannot be recognized by the APC/C^{Cdh1}. (A) Candidates of three D-box sequences (RxxL) in human Borealin protein. (B) Three D-box mutants (mt-D1, mt-D2 and mt-D3) were generated. GFP-tagged Borealin WT, mt-D1, mt-D2 or mt-D3 mutant were co-transfected with HA-tagged Cdh1 in 293T cells. Expression level of GFP-Borealin protein was examined by immunoblotting using anti-GFP antibody. Cul1 expression was used as a loading control. (C) GFP-tagged Borealin WT or all D-box mutant (mt-D1+D2+D3) was co-transfected with HA-tagged Cdh1 in 293T cells. Expression level of GFP-Borealin protein was examined by immunoblotting using anti-GFP antibody. Cul1 expression was used as a loading control.

Journal of Cell Science • Supplementary information

Figure S3

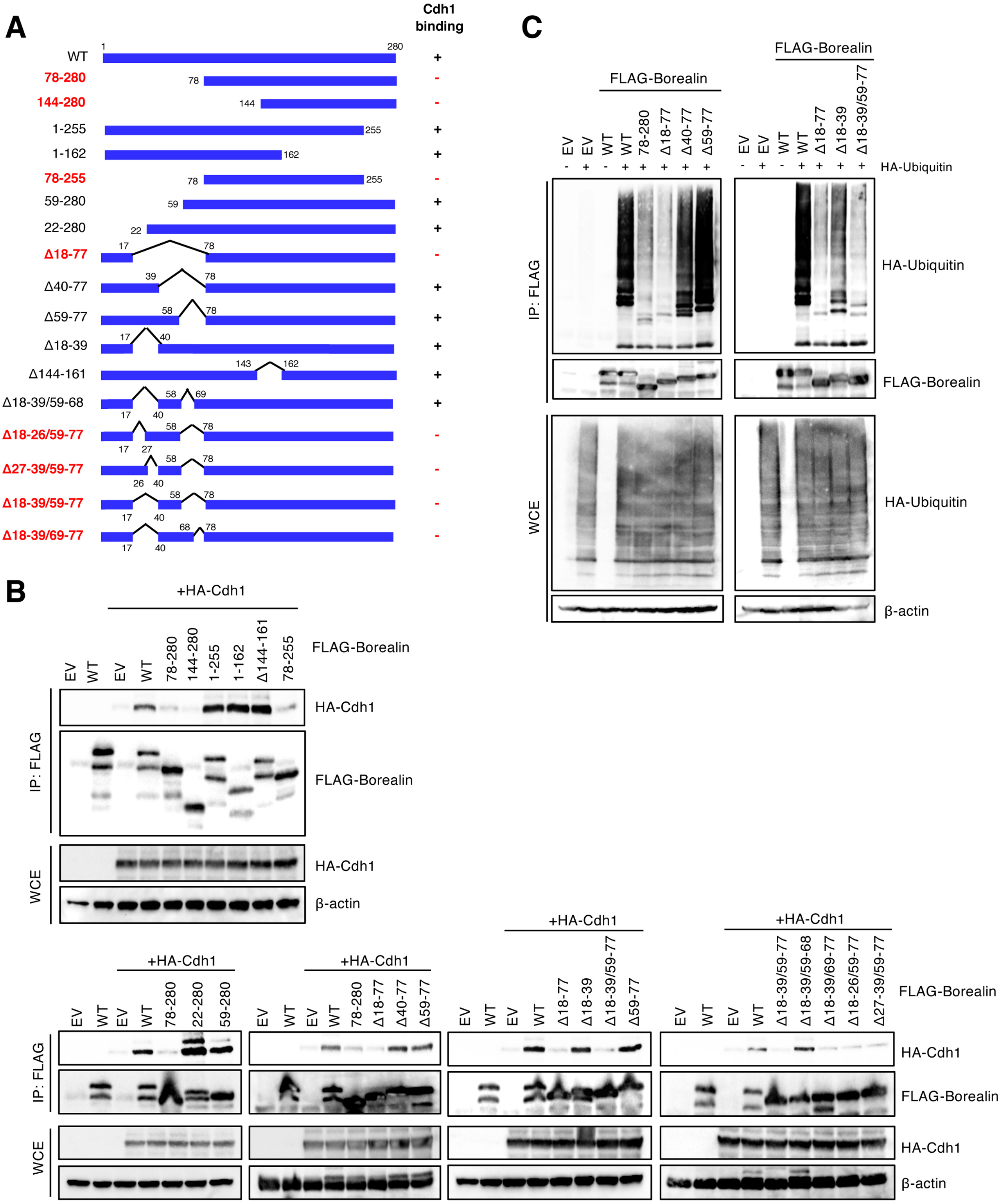

Figure S3. Mapping of the Cdh1-binding region in Borealin protein. (A) The indicated deletion mutants were generated. Results of Cdh1 binding is shown. (B) FLAG-Borealin WT or deletion mutants described in Figure 3A was co-transfected with HA-Cdh1 in 293T cells. Cell extracts were immunoprecipitated using anti-FLAG antibody. The whole cell extracts (WCE) and the immunoprecipitants (IP) were blotted with anti-HA and anti-FLAG antibodies. ß-actin expression was used as a loading control. (C) *In vivo* ubiquitylation assay was performed. EV, FLAG-tagged Borealin WT, or mutants were co-transfected with HA-tagged ubiquitin in 293T cells. Cell extracts were immunoprecipitated with an anti-FLAG antibody and blotted with an anti-HA antibody. ß-actin expression was used as a loading control.

Figure S4

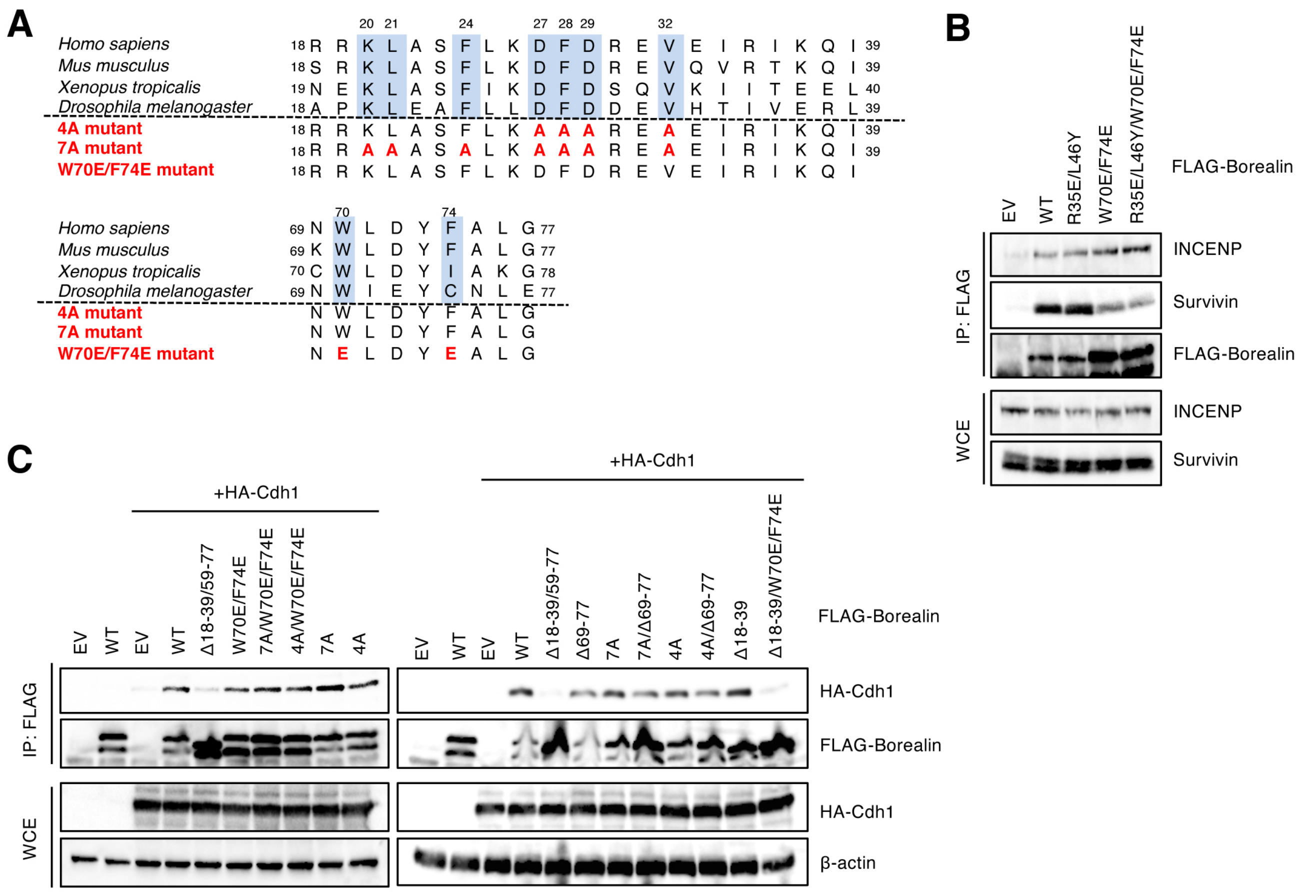

Figure S4. N-terminus region in Borealin protein are essential for Cdh1-binding. (A) Comparison of amino acid sequences of *Homo sapiens* borealin protein from 18 to 39 and 69 to 77 with *Mus musculus*, *Xenopus tropicalis*, and *Drosophila melanogaster*. As seven amino acids are highly conserved among species among residues 18-39, 4A and 7A mutants were generated. (B) HeLa cells transfected with either Borealin WT, Borealin (R35E/L46Y), Borealin (W70E/F74E) and Borealin (R35E/L46Y/W70E/F74E) were synchronized at prometaphase by using nocodazole. Cell extracts of synchronized HeLa cells were immunoprecipitated with anti-FLAG antibody, and the precipitants were blotted with anti-INCENP, anti-Survivin, and anti-FLAG antibodies. (C) FLAG-Borealin WT or indicated mutants described in Figures S4A and S5A were co-transfected with HA-Cdh1 in 293T cells. Cell extracts were analyzed by immunoprecipitation and immunoblotting using tag antibodies. ß-actin expression was used as a loading control.

Figure S5

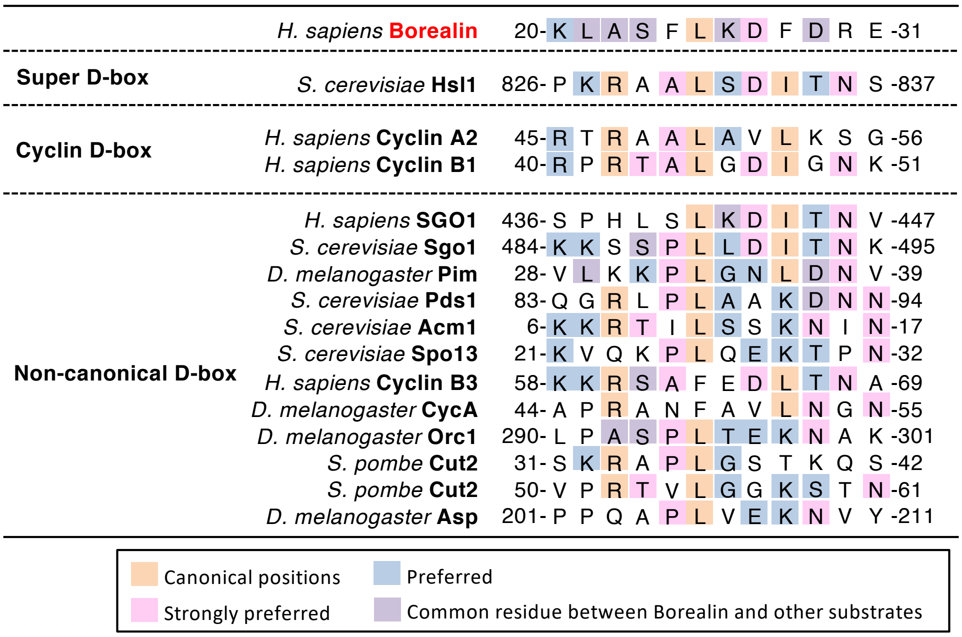

Figure S5. Comparison of D-box sequence of Borealin with that of other APC/C substrates. Davey and Morgan previously showed the preference of the D-box-binding pocket and representative experimentally validated D-box instances (Davey and Morgan, 2016). We added the D-box sequence of Borealin and modified the preference of the D-box sequences. Green indicates the consensus residues of the degron (those residues that are present in the majority of instances), pink indicates the residue with strong preferences for particular residues based on information from characterized degrons, blue indicates the residue with preferences, and yellow indicates the disfavored residues but with overlap between Borealin and other substrates.

Figure S6

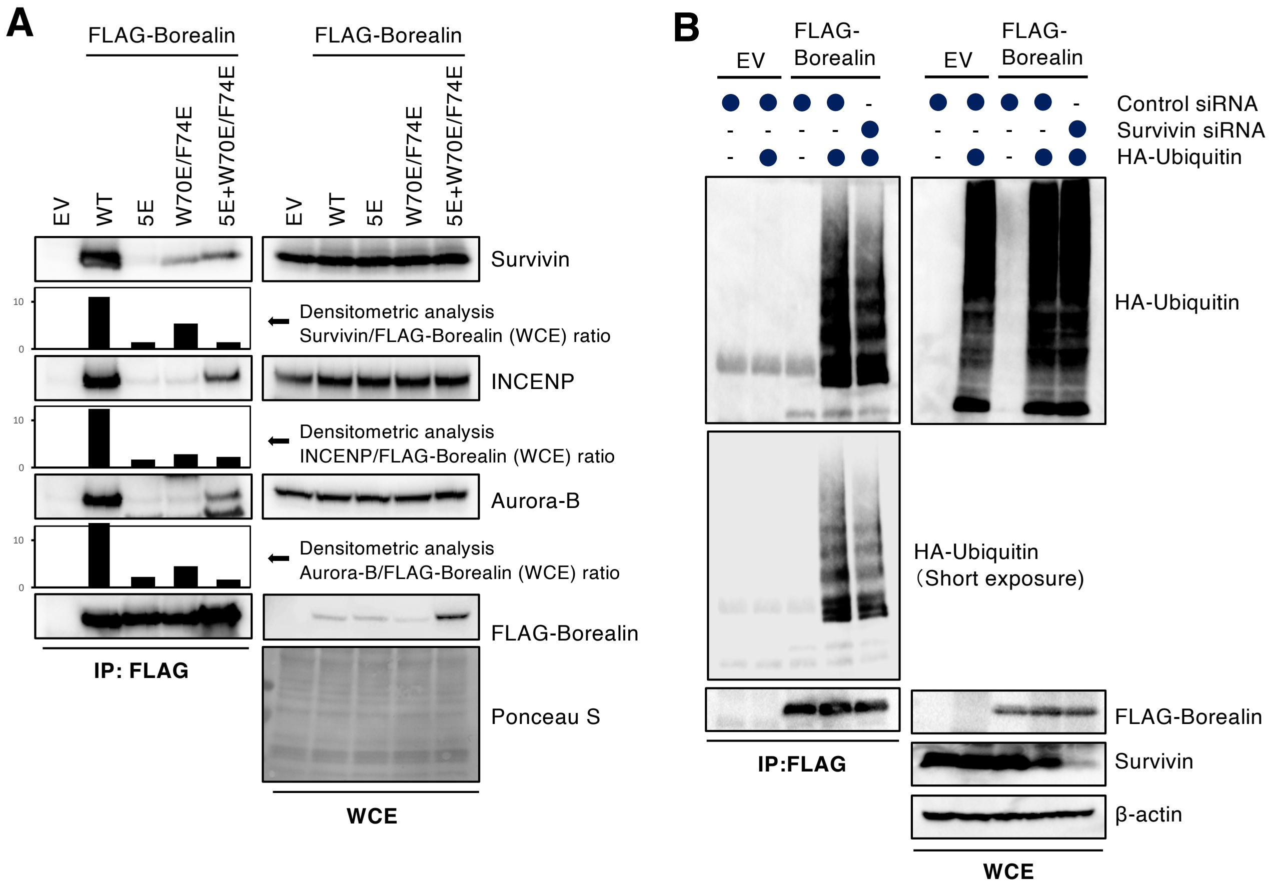

Figure S6. Survivin depletion do not interfere Borealin ubiquitylation. (A) HeLa cells transfected with FLAG-tagged Borealin WT, 5E mutant, W70E/F74E mutant, or 5E+W70E/F74E mutant were synchronized at prometaphase by using nocodazole. Cell extracts were immunoprecipitated using an anti-FLAG antibody, and blotted with anti-FLAG, anti-Survivin, anti-Aurora-B, and anti-INCENP antibodies. Ponceau S staining was shown as a loading control. Densitometric analysis of FLAG-Borealin, Aurora-B, Survivin, and INCENP expression was performed. Graph shows the Aurora-B, Survivin, or INCENP (IP)/FLAG-Borealin (WCE) ratio. (B) *In vivo* ubiquitylation assay was performed. FLAG-tagged Borealin and HA-tagged ubiquitin were co-transfected with either control and survivin siRNA in 293T cells. Cell extracts were analyzed by immunoprecipitation and immunoblotting using tag antibodies. Endogenous Survivin were also examined. ß-actin expression was used as a loading control.

Journal of Cell Science • Supplementary information
