# Supplementary material for: APC/CCdh1 is required for the termination of chromosomal passenger complex activity upon mitotic exit
Source: J Cell Sci. 2020 Sep 15;133(18):jcs251314. doi: 10.1242/jcs.251314 (PMC7520452; doi:10.1242/jcs.251314)
Supplement: Reviewer comments [file joces-133-251314_review_history.pdf]

APC/C^{Cdh1} is required for the termination of chromosomal passenger complex activity upon mitotic exit

Takaaki Tsunematsu, Rieko Arakaki, Hidehiko Kawai, Jan Ruppert, Koichi Tsuneyama, Naozumi Ishimaru, William C. Earnshaw, Michele Pagano and Yasusei Kudo
DOI: 10.1242/jcs.251314

Editor: David Glover

Review timeline
Submission to Review Commons: 16 March 2020
Submission to Journal of Cell Science: 6 July 2020
Accepted: 13 July 2020

Reviewer 1

Evidence, reproducibility and clarity

In this manuscript, Tsunematsu and colleagues reveal the APC/C cdh1 mediated ubiquitination and subsequent degradation of the chromosomal passenger complex member Borealin when cells have exited mitosis. The CPC, consisting of INCENP, survivin, Borealin, and Aurora B kinase, is important for faithful chromosome segregation during mitosis and for successful cytokinesis. The activity of this protein complex is reduced in G1 phase, most likely through regulation of the CPC protein levels, which are known to peak in mitosis and to be very low in G1. Aurora B is degraded by the proteasome in G1 after ubiquitination by APC/C cdh1, but how protein levels of the other CPC members are regulated is still not known. Here the authors demonstrate that Borealin is a target of the APC/C-cdh1 in G1 and propose that degradation of both Borealin and Aurora B contribute to timely progression through S phase.

The observation that Borealin is a target of the APC/C cdh1 is interesting and novel, but the biological implication of APC/C-cdh1-mediated degradation of Borealin in cell cycle progression remains very weak. Moreover, the authors should perform additional experiments to further address how Survivin regulates Borealin ubiquitylation and degradation.

(Other) major points:

1) Figure 2D: FoxM1 is a target of APC/C Cdh1 (Park et al, MBoC, 2008; Laoukili et al, Cell Cycle 2008) and a transcription factor for at least Aurora B , it is important to show the total levels of FoxM1 after Cdh1 depletion and to figure out to what extent stabilization of FoxM1 is contributing to the increased levels of Borealin and Aurora B (this also applies to Figures 5A and 6A). Moreover, according to these blots it seems that Survivin is also more stable after Cdh1depletion, but INCENP is not. Does this mean that INCENP is the only CPC member whose expression is not regulated by Cdh1? If INCENP levels remain low in interphase after Cdh1 depletion, how would Aurora B be activated in Cdh1 depleted interphase cells? These issues should be addressed.

2) To pinpoint when Borealin and APC/C-cdh1 interact, the IPs shown in figure 2A should be repeated in synchronized cell populations at different time points from a nocodazole release.

3) The authors have done an impressive job in mapping the Cdh1 interaction domains in Borealin. They identify two different regions that are both required. Only deletion of both regions affects the interaction with Cdh1 based on co-IP experiments (Fig S3). The prediction from this

© 2020. Published by The Company of Biologists under the terms of the Creative Commons Attribution License (https://creativecommons.org/licenses/by/4.0/).

analysis is that deletion of a single region might already affect binding affinity. However, this can only be assessed by in vitro binding studies with recombinant proteins. This maybe a lot to ask. However, the following points should be addressed. It is puzzling that ubiquitylation of the Borealin-3E mutant is reduced, whilst it can still interact with HA-cdh1 (figure 3B, C). Since the authors explore the mechanistic details of the APC/C Cdh1-mediated degradation of Borealin (p.5), it is important to find an explanation for these observations. Could the 3E mutations block the nearby lysine residues (for instance K26) from being ubiquitylated? How stable is this 3E mutant compared to WT and Borealin 5E? Can it bind Survivin? If the Borealin 3E mutant still binds survivin, it might be a better choice to study protein stability and the functional implication of Borealin ubiquitylation in cell cycle progression, than the 5E mutant that does not interact with Survivin.

4) Borealin 5E is still degraded after noco release (Figure 3E). How?

5) Is there any mitotic or cell cycle defect observed when endogenous Borealin is replaced by Borealin 3E, Borealin 5E (siRNA knock-down and add-back experiment)?

6) The authors show that a Borealin-5E mutant does not interact with Cdh1 and that its ubiquitylation is significantly reduced (Figure 3B, C). However, due to the W70/F74E mutations, its binding to Survivin is also impaired (Fig. S4B, Jeyaprekash et al, Cell 2007). Previous work from amongst others Klein et al. (MBoC, 2006) has demonstrated that knock- down of Survivin by siRNA has a dramatic effect on the protein levels of Borealin: the presence of Survivin somehow affects protein stability of Borealin. The authors may have a lead to explain these older observations but unfortunately, they fail to thoroughly investigate and discuss the contribution of Survivin to Borealin protein stability. In figures 4 and S6, the authors show that overexpression of survivin reduces the ubiquitylation of flag-borealin, whilst knock-down of endogenous survivin does not have a clear effect on Flag-Borealin ubiquitination. The observation that Survivin, but not Survivin dE3, overexpression interferes with the ubiquitination of Flag-Borealin is potentially very interesting as it suggests that the binding of Survivin to Borealin somehow regulates its ubiquitination, and thereby maybe its stability. The authors should test a number of (obvious) things:

- Do Survivin and Cdh1 compete in binding Borealin? In other words, can overexpressed Flag-Borealin still interact with Cdh1 in the presence of overexpressed Survivin?
- Is ubiquitination of the flag-Borealin W70/F74E mutant reduced by overexpression of Survivin? If not, it would be in line with the idea that when bound to Survivin, Borealin is less accessible for APC/C cdh1 mediated ubiquitination.
- How stable is the Borealin W70/F74E mutant compared to WT Borealin in a Borealin knock-down/add-back situation?
- Based on the effect of overexpressed Survivin, the prediction would be that knock-down of endogenous Survivin would enhance the ubiquitination of Borealin, but this is not observed in figure S6. However, since the assays were performed with overexpressed Flag-Borealin it could be that the fraction of overexpressed flag-Borealin that is ubiquitinated, is the fraction that is not bound to endogenous Survivin. Alternatively, or additionally, the ubiquitination assay might be saturated at the timepoint the cell lysates were prepared. The authors should sort these things out.

7) The Western Blot in figure 5A is of poor quality, making it difficult to interpret. It should be repeated. Aurora B and Borealin seem to be up-regulated already at time point 0, whilst the authors claim these proteins are more stable at later time points in Fzr-/- MEFs. Furthermore, in contrast to Figures 1A,2D, the INCENP levels remain stable throughout the cell cycle. Is this a feature of MEFs vs HeLa cells or does the band maybe not correspond to mouse INCENP? The authors should show this band represents INCENP by knocking down mouse INCENP. Moreover, since p-T232 levels also depend on the total protein level of Aurora B, it may be better to use a different read-out for Aurora B activity, such as for instance H3S28ph. Moreover, as mentioned earlier, as FOXM1 is a target of APC/C cdh1, the total levels of FOXM1 should be shown as well.

8) The conclusion that "sustained CPC activity induces early DNA replication" is not supported by the data (figure 5B). In Fzr-/- cells, DNA replication starts earlier compared to WT cells (figure 4B). However, in the presence of the Aurora B kinase inhibitor, Barasertib, replication starts at the same timepoint as in the non-treated Fzr-/- cells. The only difference is at t=24 where the % of Edu positive cells drops in the Fzr-/- MEFS but is still high when Barasertib is present. This could mean that inhibition of Aurora B activity might prolong S phase in Fzr-/- cells. Moreover, the percentage

© 2020. Published by The Company of Biologists under the terms of the Creative Commons Attribution License (https://creativecommons.org/licenses/by/4.0/).

of EdU positive cells only reflects the proportion of cells that are in S phase at a certain moment, and not replication efficiency (as suggested on page 8). To get a better idea of the replication efficiency, replication speed should be measured in Fzr-/- and WT cells +/- Barasertib using IdU/ClU pulses. Or the Edu data should be better explained and interpreted.

9) To test if Aurora B is active in Fzr-/- interphase cells, the authors perform IF for H3S10ph. First, interphase is not just G1 as suggested on page 8, but refers to G1/S/G2 (all phases except M phase). Second, it is surprising that interphase nuclear foci of Aurora B and H3S10ph were not observed in the Fzr+/+ MEFs (page 8). In many cell types Aurora B and H3S10ph foci become visible in late S phase and G2 (see for example: Ruppert et al, EMBO J (2018)). The authors should quantify the number of Aurora B/H3S10ph positive nuclei within the population of Cyclin A+ and Cyclin A- cells. It is expected that in the Fzr-/- MEFs this fraction is specifically increased in the Cyclin A-, and less so in the Cyclin A+ cells.

10) The high interphase levels of CPC proteins in the teratoma cell lines is interesting, but some functional consequence of having these high CPC levels in interphase (that can be (partially) reversed by ABK kinase inhibition) should be shown. Moreover, the authors should do a better job in showing that these high levels are indeed a consequence of Emi overexpression and thus APC/C cdh1 inhibition and increased protein stability. They should knock-down Emi1 (FigS8) and repeat the TB+RO release shown in 6A. Are the levels of Borealin and Aurora B now going down similar as in somatic (Hela) cells? And what happens to INCENP levels which is not a target of APC/C cdh1 according to figure 2D? In addition, as shown in figure S8C, mRNA levels of CPC members also appear to be high in these pluripotent stem cells. To assess the contribution of transcription/translation and protein stability to the high interphase CPC protein levels in these cells, the authors should test to what extent cycloheximide treatment affects protein levels of CPC proteins in these cells.

11) In figure 6D the authors show IF images of endogenous Aurora B, INCENP and Survivin in NCC-IT-A3 interphase cells. It is unclear why Flag-Borealin is overexpressed in these cells and if this Borealin overexpression could somehow affect the levels of the other CPC members. It is important to perform IFs for endogenous Aurora B, INCENP, Survivin (and preferentially endogenous Borealin) without exogenously expressed flag-Borealin. The levels should be compared to the levels in interphase HeLa cells by mixing the two cell lines. One of the two cell lines can be labelled through stable expression of fluorescently tagged H2B. By performing IFs for CPC proteins and Cyclin A on co-cultured HeLa and NCC-IT-A3 cells, the CPC protein levels in cyclin A+ (HeLa vs NCC-IT-A3) vs cyclin A-(HeLa vs NCC-IT-A3) cells can be quantified by quantitative IF.

12) In general, the discussion section is not very clear or logical. A few examples: page 10/11: "Previous reports show that.....additional binding affinity for the APC/C.
Therefore, Borealin may require residues W70 and W74 that are essential for the interaction with Survivin for additional binding affinity to Cdh1. However, Survivin does not act as an adaptor of Borealin ubiquitylation". >>> A conclusion or model explaining their findings would be helpful here.

page 11/12: "Surprisingly, the forming the CPC for Aurora B activity is not always necessary. Upon mitotic exit, APC/C cdh1-mediated degradation of Borealin and/or Aurora B might trigger the termination of CPC function and Aurora B activity. Indeed, we demonstrated that the Aurora B and Borealin could form CPC complex in interphase of EC cells with low activity of APC/C cdh1". I am unable to grasp the point/ rationale of this part of the discussion.

Finally, the part on CPC overexpression, carcinogenesis and the role of Cdh1 in this (page 12) is a bit overstated and not supported by any of the data presented in the manuscript

Minor comments:

Figure 1: The authors have indicated timepoint 12hrs as "S phase", it is unclear on what marker this is based. In other words, how do the authors know this is indeed the moment these Hela cells enter S phase?
Figure 2A (right panel): the lanes of Myc-Cullin do not match the rest of the blot (8 lanes in Myc-Cullin IP, whereas there are 7 lanes in the other rows). In addition, Myc-CUL2 was not expressed,

© 2020. Published by The Company of Biologists under the terms of the Creative Commons Attribution License (https://creativecommons.org/licenses/by/4.0/).

and hence it cannot be excluded that CUL2 is involved in the degradation of borealin. This WB needs to be repeated.

It would be better to show the effects of Cdh1 overexpression on endogenous Borealin levels instead of overexpressed GFP-Borealin as shown in figure 2C. This is actually shown in U2OS with Dox-inducible cdh1 in figure S1B, this figure can be part of the main figure.

Figure 2E: A control for functionality of the cdc20 knock-down is lacking (for instance lack of Ub of cyclin B). Moreover, there is still significant ubiquitination of flag-Borealin in the siCdh1 condition. Is this due to poor knock-down of Cdh1 or does cdc20 contribute? Would co- depletion of cdh1 and cdc20 further reduce the ubiquitination of Flag-Borealin?

Figure 3E and 3F are redundant, and 3F is more convincing than 3E as it shows Flag- Borealin 5E stabilization is not affected by translation rate. Figure 3E may be be shown as a supplemental figure.

Materials and methods:

It would help to mention from what kind of tissues PA1, MRC5 and NCC-IT-A3 were originally derived.
Where do all the different plasmids encoding Borealin come from? Please include references.
The authors should mention whether the Fzr-/- MEFS were derived from conditional or constitutive FZR knock-out mice.

Significance

The observation that Borealin is a target of the APC/C cdh1 is interesting and novel, but the biological implication of APC/C-cdh1-mediated degradation of Borealin in cell cycle progression remains very weak. Moreover, the authors should perform additional experiments to further address how Survivin regulates Borealin ubiquitylation and degradation, as it may explain earlier observations showing that Survivin depletion also affects Borealin protein levels.

Reviewer 2

Evidence, reproducibility and clarity

In the manuscript from Tsunematsu, Kudo, and colleagues, the authors examine the cell cycle-regulated degradation of the chromosomal passenger complex, with emphasis on the Borealin subunit. They conclude that Borealin, which contributes to various processes in mitosis, is degraded in the subsequent G1 phase by targeting the APC/C ubiquitin ligase via Cdh1. They further conclude that degradation of Borealin and Aurora B by APC/C-Cdh1 is necessary to prevent CPC activity in G1 and to promote DNA replication in S phase. The data on Borealin degradation by Cdh1 is robust and thorough, with a strong combination of in vivo and in vitro experiments. The evidence for excess CPC activity in G1 is not yet convincing, but could be made much stronger with additional quantification of the experiments. The conclusion that excess CPC activity affects the entry into S-phase is not justified by the data currently presented.

Major comments:

1. I have a number of issues with the interpretation of the S phase entry experiment shown in Figure 5B. Firstly, the figure legend does not contain any information on how many times the experiment was conducted and what the error bars represent. Second, what is the rationale behind the peak value measuring the "efficiency of DNA replication"? Surely, this is not a measure of the total number of cells that complete S phase, as some of the cells are likely to have already completed S phase at the peak point and the actual peak may be missed if it is in between time points. Third, the effect of Barasertib on the FZR1-deleted cells appears to be a delay in S-phase completion, not a decrease in the number of cells that initiate replication early as claimed by the authors (the 3-21 hour time points are unchanged). With these three issues, the claim that

© 2020. Published by The Company of Biologists under the terms of the Creative Commons Attribution License (https://creativecommons.org/licenses/by/4.0/).

"abnormal DNA replication in Fzr-/- MEFs was rescued by Aurora-B kinase inhibitor treatment" in the discussion is unfounded.

2. The microscopy in the manuscript requires quantification (Supplementary Figure 7 A-C and Figure 6D). Single anecdotal examples are not enough to inform the reviewer of how robust the results are. The percentage of cells with H3S10 foci should be measured in each experiment, preferably with 3 biological replicates and statistical significance.

3. With the Borealin 5E mutant, the protein levels are greatly increased even at cell cycle stages where Cdh1 should not be active (t = 0 in Figure 3E and Figure 4A). Furthermore, the levels decrease after G1 entry to a similar extent to the wildtype Borealin. These data suggest that the increase in expression of the 5E mutant is not due to preventing Cdh1-targeted APC/C activity.

Minor comments:

1 In Figure 2A, there appears to be an extra lane in the Myc-Cullin IP blot. It looks like there are 6 lanes in line with the other blots that have 5 lanes in the same width of blot.

2 It would strengthen the argument of a direct interaction between Borealin and Cdh1 if the pull-down with reconstituted protein were repeated with the 5E mutant.

3 Near the bottom of page 4, the authors claim "Borealin levels are low in G1 and rise during late S, G2, and M." However, the data cited for this (Figure 1A) only shows the M to G1 transition.

4 In Figure 1C, why do the authors use two general proteosome inhibitors, but not the more specific APC/C inhibitor proTAME?

5 . In Figure 2E, when the ubiquitination is decreased with Cdh1 depletion, why are the overall levels FLAG-Borealin not increased? Is this a result of overexpression?

6 The rational for the design of the experiment shown in Figure 3F is not explained in the text.

7 In supplementary figure S8C, it is not clear that the Aurora B levels decrease after Emi1 inhibition as claimed in the text.

8 The data in Figure 2D shows that INCENP levels decrease in G1 even when Cdh1 is inhibited. How do the authors explain the Aurora B activity observed in G1 upon FZR1 deletion when the essential kinase activator is still missing at this stage?

Significance

Given that the degradation of Aurora B by Cdh1 has been previously reported, the additional knowledge that the Borealin subunit of the CPC is regulated in a similar way will not provide a major advance in the field. Furthermore, the lack of convincing phenotypes as this stage of the manuscript (see major comments 1 and 2) also diminishes the impact of the study.
However, many of the experiments appear to be well done and are certainly worth publishing.

Reviewer 3

Evidence, reproducibility and clarity

The chromosomal passenger complex (CPC), containing the mitotic kinase Aurora B and the non-enzymatic subunits Boralin, Surivin and Incenp, controls multiple aspects of cell division. How the activity of the CPC is regulated is therefore an important area of research. Tsunemato and colleagues analyse the degradation of the CPC at the end of mitosis. They focus on the Borealin subunit and find that Borealin protein stability is controlled by APC/C-Cdh1. They identify a non-canonical D-box in Borealin that is required for Borealin ubiquitiniation by APC/C-Cdh1. Access to

© 2020. Published by The Company of Biologists under the terms of the Creative Commons Attribution License (https://creativecommons.org/licenses/by/4.0/).

this degradation motif seems to be limited by Survivin binding to Borealin suggesting that there is negative relationship between CPC complex formation and degradation of its components. The authors then investigate the potential consequences of impaired CPC degradation. In Fzr1-/- (gene for Cdh1) mouse embryonic fibroblasts the protein levels of the CPC components are stabilised at the end of mitosis. When synchronised, these cells enter S-phase prematurely, an effect that can be partially alleviated by treating the cells with Aurora B inhibitor. The authors thus conclude that untimely high levels of CPC early in the cell cycle dysregulate DNA replication. The authors then analyse pluripotent stem cells which have much lower levels of APC/C activity than somatic cells. Consequently, the levels of the CPC are much higher in these cells, and Histone H3-Ser10 phosphorylation, a read-out for Aurora B activity, can be observed even in interphase in these cells.

Overall, the study is well conducted, and the data shown largely support the conclusions drawn in the text. What is not quite so well worked out, are the functional consequences of untimely stable CPC. This is the aspect of the manuscript that has to be improved. It is not quite clear why the authors decide to study the consequences of not having Cdh1 in Fzr1-/- MEFs instead of performing RNAi in human cells. They come to the conclusion that cells lacking Cdh1 enter S-phase prematurely (as previously described), and that this is due to the presence of CPC activity too early in the cell cycle. This conclusion is supported by the fact that the premature entry into S-phase can be partially alleviated by treating the cells with the Aurora B inhibitor Barasertib. However, this experiment does not provide any direct evidence that the CPC is directly involved in the regulation of S-phase. Loss of Cdh1 is expected to derail many aspects of cell cycle control, and the fact that inhibition of Aurora B, another mitotic enzyme with many targets, alleviates the loss of Cdh1, does not necessarily mean that Aurora B directly promotes early S-phase, and to my knowledge, there is no evidence that the CPC in any way regulates S-phase entry. A better experiment would be to use human HeLa Flp-In cells to conduct an RNAi rescue experiment and replace the endogenous Borealin with the Borealin mutant that cannot be ubiquitinated and then ask what the functional consequences are of this situation. If again, a premature entry into S- phase is observed, it could then be checked how this affects the rest of the cell cycle. Do you get DNA-bridges, indicative of incomplete replication?
The last experiment in the manuscript seems like an unnecessary add-on, the experiments in the pluripotent stem cells do not come to any clear conclusions and would better be left out.

Minor comments:

The title: The title should be changed. The authors do not provide any clear evidence that CPC activity is maintained when the CPC is not degraded.

Figure 2: the Myc-Cullin 2 expression is very poor.

Figure 3: The rationale for choosing the residues for mutational analysis is not clearly explained.

Figure 3E: Since the mutated Borealin is expressed at a higher level, should the ratio of Flag-Borealin/actin not be higher for the 5E mutant? Or have both WT and 5E ratios at 0h of Noc release been normalised to 1?

Figure 4B: The result in Figure 4B should be quantitated.

Figure 5A: Can the authors provide a nicer blot for survivin? IF images would be really useful to back up the claim that the CPC is not degraded in the absence of Cdh1/Fzr1. The IF images from S7 could be put into figure 5.

Figure 5B: Could the authors show representative images in addition to the graph in Figure 5B? What about subsequent changes to cell cycle progression, e.g. compromised chromosome segregation, e.g. DNA bridges?

Figure 6D: Why do the authors look at overexpressed Flag-Borealin instead of the endogenous Borealin?

© 2020. Published by The Company of Biologists under the terms of the Creative Commons Attribution License (https://creativecommons.org/licenses/by/4.0/).

Significance

The question of how the chromosomal passenger complex is regulated is a key question in the cell cycle field. Any information about the regulation of the different CPC subunits is therefore important. This manuscript provides interesting new information but the functional consequences of Borealin mis-regulation have not yet been sufficiently worked out.

Author response to reviewers' comments

➢ **Initial response/ Revision plan**

As suggested by Reviewer #3, we removed the last experiments with embryonal carcinoma cells (Figure 6 and Supplementary Figure S8).

Reviewer #1

This Reviewer is enthusiastic about our results stating that: *"The observation that Borealin is a target of the APC/C cdh1 is interesting and novel"*. He/she asks that we address the following specific issues. We appreciate that the referee is enthusiastic about certain aspects of our studies, but it is clear that if we were to do all of the experiments proposed here, it would take years. These are good ideas for future studies, but in the context of the present MS, we feel that many of them can be classed as "Reviewer Experiments" (see Ploegh, Nature: PMID: 21525890) and we hope that we will be allowed to respond to a reasonable selection of them.

Major points:

1. *Figure 2D: FoxM1 is a target of APC/C Cdh1 (Park et al, MBoC, 2008; Laoukili et al, Cell Cycle 2008) and a transcription factor for at least Aurora B , it is important to show the total levels of FoxM1 after Cdh1 depletion and to figure out to what extent stabilization of FoxM1 is contributing to the increased levels of Borealin and Aurora B (this also applies to Figures 5A ~~and 6A~~). Moreover, according to these blots it seems that Survivin is also more stable after Cdh1 depletion, but INCENP is not. Does this mean that INCENP is the only CPC member whose expression is not regulated by Cdh1? If INCENP levels remain low in interphase after Cdh1 depletion, how would Aurora B be activated in Cdh1 depleted interphase cells? These issues should be addressed.*

- As suggested by this Reviewer, we will evaluate the total levels of Fox1 in Cdh1 depleted cells and *FZR1*$^{-/-}$ MEFs (Figures 2D and 5A).
- This Reviewer pointed out that INCENP levels remained low in interphase after Cdh1 depletion. As shown in the Attached Figure 1, in a longer exposure of the blot, INCENP expression can be detected even in G1. Therefore, Aurora-B may be activated by remaining low levels of INCENP. To better explore this possibility, we will examine the nuclear foci of Borealin, Aurora-B, and INCENP in G1 phase of Cdh1 depleted cells by immunofluorescence.

2. *To pinpoint when Borealin and APC/C-cdh1 interact, the IPs shown in figure 2A should be repeated in synchronized cell populations at different time points from a nocodazole release.*

- As suggested by this Reviewer, we will examine the interaction between Cdh1 and **Borealin in G1 and M phase.**

3. *The authors have done an impressive job in mapping the Cdh1 interaction domains in Borealin. They identify two different regions that are both required. Only deletion of both regions affects the interaction with Cdh1 based on co-IP experiments (Fig S3). The prediction from this analysis is that deletion of a single region might already affect binding affinity. However, this can only be assessed by in vitro binding studies with recombinant proteins. This maybe a lot to ask. However, the following points should be addressed. It is puzzling that ubiquitylation of the Borealin-3E mutant is reduced, whilst it can still interact with HA-cdh1 (figure 3B, C). Since the authors explore the mechanistic details of the APC/C Cdh1-mediated degradation of Borealin (p.5), it is important to find an explanation for these observations. Could the 3E mutations block the nearby lysine residues (for instance K26) from being ubiquitylated? How stable is this 3E*

© 2020. Published by The Company of Biologists under the terms of the Creative Commons Attribution License (https://creativecommons.org/licenses/by/4.0/).

mutant compared to WT and Borealin 5E? Can it bind Survivin? If the Borealin 3E mutant still binds survivin, it might be a better choice to study protein stability and the functional implication of Borealin ubiquitylation in cell cycle progression, than the 5E mutant that does not interact with Survivin.

- **We would like to thank the Reviewer for an interesting suggestion. We generated mutants of the nearby lysine residues (K20 and K26) and then examined the ubiquitylation. However, K20R, K26R, and K20R/K26R mutants did not affect the ubiquitylation (please see Attached Figure 2). Therefore, these lysine residues may either not be involved in ubiquitylation of Borealin or, as it is often the case, ubiquitylation may switch to other lysines if they are removed.**

- **To find the Borealin degron, we generated several deletion mutants (Supplementary Fig. S3A) and evaluated the binding of these mutants to Cdh1. This analysis revealed that the residues 18-39 and 69-77 were required for Cdh1 binding and ubiquitylation. The residues, W70 and F74 are essential for Cdh1 binding (Supplementary Fig. S4C). The conserved hydrophobic residues, F24, L25, and F28 are also essential for Cdh1 binding. As shown in the above experiment, the nearby lysine residues (K20 and K26) within this region are not involved in the ubiquitylation. Again, we carefully checked the sequence of the residues 18-39 including the conserved hydrophobic L21 and V32 residues. As a result, we generated a new 5E mutant (L21E/F24E/L25E/F28E/V32E) by mutating all five conserved hydrophobic residues in this region (please see Attached Figure 3). All five residues are important for the interaction of Borealin with Survivin. Moreover, we also generated 5E+W70E/F74E mutant. Then, we examined the ubiquitylation of these new Borealin mutants. Interestingly, the Borealin 5E+W70E/F74E mutant remarkably suppressed *in vivo* ubiquitylation, compared to 3E, 3E+W70E/F74E (previous 5E mutant), and 5E (please see Attached Figure 4A). We would like to thank the Reviewer for a valuable comment. So, we will focus on the 5E+W70E/F74E mutant for further experiments. Given these new results, we replaced the Figures 3A and 3C. Moreover, we repeated the Figure 3B (please see Attached Figure 4B). The binding of 5E+W70E/F74E mutant with Cdh1 was substantially reduced, compared to wild type, the W70E/F74E mutant, and the 5E mutant. We will replace the Figure 3B, 3D, 3E, and 4A after repeating the experiments using Borealin 5E+W70E/F74E mutant.**

4. *Borealin 5E is still degraded after noco release (Figure 3E). How?*

- As described above, we will now focus on the new Borealin 5E+W70E/F74E mutant. We will check its levels after nocodazole release. We expect that the 5E+W70E/F74E mutant will not be degraded in G1, because ubiquitylation of 5E+W70E/F74E mutant is significantly suppressed. The original Borealin 5E mutant (we now call this mutant 3E+W70E/F74E) is instead still ubiquitylated, explaining why it was still degraded after nocodazole release.

5. *Is there any mitotic or cell cycle defect observed when endogenous Borealin is replaced by Borealin 3E, Borealin 5E (siRNA knock-down and add-back experiment)?*

- **Considering the above comments #3 and #4 and the suggestion by this Reviewer, we will examine mitotic and cell cycle defects after replacement of endogenous Borealin by wild type and the 5E+W70E/F74E mutant (siRNA knock-down and add-back experiment). For the add-back experiment, we used a single vector for co-expressing Borealin and its shRNA as shown in Attached Figure 5. In our preliminary experiment, Borealin shRNA induced an increase in cell size. Wild type Borealin and the W70E/F74E mutant, but not the 5E+W70E/F74E mutant rescued this phenotype. We will investigate the detailed phenotypes, including mitotic and cell cycle defects.**

6. *The authors show that a Borealin-5E mutant does not interact with Cdh1 and that its ubiquitylation is significantly reduced (Figure 3B, C). However, due to the W70/F74E mutations, its binding to Survivin is also impaired (Fig. S4B, Jeyaprekash et al, Cell 2007). Previous work from amongst others Klein et al. (MBoC, 2006) has demonstrated that knock-down of Survivin by siRNA has a dramatic effect on the protein levels of Borealin: the presence of Survivin somehow affects protein stability of Borealin. The authors may have a lead to explain these older observations but unfortunately, they fail to thoroughly investigate and discuss the contribution of Survivin to*

© 2020. Published by The Company of Biologists under the terms of the Creative Commons Attribution License (https://creativecommons.org/licenses/by/4.0/).

Borealin protein stability. In figures 4 and S6, the authors show that overexpression of survivin reduces the ubiquitylation of flag-borealin, whilst knock-down of endogenous survivin does not have a clear effect on Flag-Borealin ubiquitination. The observation that Survivin, but not Survivin dE3, overexpression interferes with the ubiquitination of Flag-Borealin is potentially very interesting as it suggests that the binding of Survivin to Borealin somehow regulates its ubiquitination, and thereby maybe its stability. The authors should test a number of (obvious) things:

- *Do Survivin and Cdh1 compete in binding Borealin? In other words, can overexpressed Flag-Borealin still interact with Cdh1 in the presence of overexpressed Survivin?*
- *Is ubiquitination of the flag-Borealin W70/F74E mutant reduced by overexpression of Survivin? If not, it would be in line with the idea that when bound to Survivin, Borealin is less accessible for APC/C cdh1 mediated ubiquitination.*
- *How stable is the Borealin W70/F74E mutant compared to WT Borealin in a Borealin knock-down/add-back situation?*
- *Based on the effect of overexpressed Survivin, the prediction would be that knock-down of endogenous Survivin would enhance the ubiquitination of Borealin, but this is not observed in figure S6. However, since the assays were performed with overexpressed Flag-Borealin it could be that the fraction of overexpressed flag-Borealin that is ubiquitinated, is the fraction that is not bound to endogenous Survivin. Alternatively, or additionally, the ubiquitination assay might be saturated at the timepoint the cell lysates were prepared. The authors should sort these things out.*

- As suggested, we examined whether the ubiquitination of the FLAG-Borealin W70/F74E mutant can be reduced by overexpressing Survivin. However, Survivin overexpression could also suppress the ubiquitylation of the Borealin W70E/F74E mutant (please see Attached Figure 6A). Although the W70E/F74E mutant shows less binding to Survivin, a small amount of Survivin still binds to Borealin. As shown in above (comment #3), there are several Survivin-binding residues within the region 18-39 and 69-77 of Borealin. Indeed, a small amount of Survivin could bind with Borealin W70E/F74E mutant via other residues. Therefore, ubiquitylation of the W70/F74E mutant may be caused by Survivin overexpression. We added this data in Supplementary Figure S6D and “Results” section.

As described above (comment #3), the Borealin 5E+W70E/F74E mutant is not ubiquitylated, when compared to 5E and W70E/F74E mutants on their own. In this experiment, we checked the binding of Borealin with Survivin. Interestingly, Borealin ubiquitylation well correlated well with its binding to Survivin (please see Attached Figure 6B), suggesting that Borealin ubiquitylation may depend on its interaction with Survivin. Indeed, the Borealin 5E+W70E/F74E and Δ18-77 mutants, which are not ubiquitylated, did not bind with Survivin. Together, these findings suggest that Cdh1 may recognize mainly Borealin in a complex with Survivin, rather than free Borealin.

However, overexpression of Survivin wild type, but not ΔEx3 mutant that does not bind Borealin, suppressed Borealin ubiquitylation. To solve this contradiction, we checked the localization of Survivin (please see Attached Figure 7). Wild type Survivin mainly localized to the cytoplasm, while the Survivin ΔNES mutant mainly localized to the nucleus (we added this data in Supplementary Figure S6C). This finding suggests that Survivin overexpression may suppress Borealin ubiquitylation by translocating it to the cytoplasm. To demonstrate this hypothesis, we will examine the effect of the Survivin ΔNES mutant (which is localized in the nucleus) on Borealin ubiquitylation. If our hypothesis is correct, the Survivin ΔNES mutant will enhance Borealin ubiquitylation.

In Supplementary Figure S6A, Survivin knockdown suppressed Borealin ubiquitylation.

In that experiment, we performed densitometric analysis to quantify the Borealin ubiquitylation. Survivin knockdown suppressed Borealin ubiquitylation by about 30% (please see Attached Figure 8). This finding is consistent with our hypothesis.

In summary, previous and present findings suggest that Survivin promotes Borealin ubiquitylation. To demonstrate this hypothesis, we will perform the following experiment:

1. We will investigate the binding of wild type Survivin, the ΔNES mutant, and the ΔEx3 mutant with Borealin by IP and GST-pull down assays. Moreover, we will check the localization of Survivin and Borealin. As shown above, wild type Survivin localized to the cytoplasm, while the ΔNES mutant localized to the nucleus. The ΔEx3 mutant also localized to the nucleus (Caldas et al.,

© 2020. Published by The Company of Biologists under the terms of the Creative Commons Attribution License (https://creativecommons.org/licenses/by/4.0/).

Oncogene 24: 1994-2007, 2005).
Therefore, we speculate that suppression of Borealin ubiquitylation may be caused by its Survivin-mediated translocation into the cytoplasm. If this speculation is true, the ΔNES mutant may enhance Borealin ubiquitylation.

2. We speculate that Survivin promotes Borealin ubiquitylation. We will evaluate if Survivin depletion influences the interaction between Borealin and Cdh1.

3. We will perform *in vitro* binding assays (WT Borealin or mutant/Cdh1, WT Survivin or ΔEx3/Cdh1, and WT Borealin or mutant/WT Survivin or ΔEx3/Cdh1).

7. The Western Blot in figure 5A is of poor quality, making it difficult to interpret. It should be repeated.

- **As suggested by this Reviewer, we will repeat the Western blot for Figure 5A.**

Aurora B and Borealin seem to be up-regulated already at time point 0, whilst the authors claim these proteins are more stable at later time points in Fzr-/- MEFs.

- **As the pointed out by this Reviewer, the expression levels of Borealin and Aurora-B were higher at time point 0 in *Fzr1$^{-/-}$* MEFs. Therefore, we changed the sentence to: "As expected, levels of Borealin and Aurora-B oscillated in *Fzr1$^{+/+}$* MEFs but were more stable throughout the cell cycle in *Fzr1$^{-/-}$* MEFs (Fig. 5A)".**

Furthermore, in contrast to Figures 1A,2D, the INCENP levels remain stable throughout the cell cycle. Is this a feature of MEFs vs HeLa cells or does the band maybe not correspond to mouse INCENP? The authors should show this band represents INCENP by knocking down mouse INCENP. Moreover, since p-T232 levels also depend on the total protein level of Aurora B, it may be better to use a different read-out for Aurora B activity, such as for instance H3S28ph. Moreover, as mentioned earlier, as FOXM1 is a target of APC/C cdh1, the total levels of FOXM1 should be shown as well.

- **The INCENP levels remain stable throughout the cell cycle in contrast to Figures 1A and 2D. We will repeat this experiment by using a second antibody that specifically recognizes mouse INCENP in Figure 5A. As suggested by this Reviewer, in this experiment, we will also check the expression of H3S28ph and FOXM1.**

8. The conclusion that "sustained CPC activity induces early DNA replication" is not supported by the data (figure 5B). In Fzr-/- cells, DNA replication starts earlier compared to WT cells (figure 5B). However, in the presence of the Aurora B kinase inhibitor, Barasertib, replication starts at the same timepoint as in the non-treated Fzr-/- cells. The only difference is at t=24 where the % of Edu positive cells drops in the Fzr-/- MEFS but is still high when Barasertib is present. This could mean that inhibition of Aurora B activity might prolong S phase in Fzr-/- cells. Moreover, the percentage of EdU positive cells only reflects the proportion of cells that are in S phase at a certain moment, and not replication efficiency (as suggested on page 8). To get a better idea of the replication efficiency, replication speed should be measured in Fzr-/- and WT cells +/- Barasertib using IdU/ClU pulses. Or the Edu data should be better explained and interpreted.

- **As suggested by this Reviewer, the finding in Figure 5B possibly indicates that inhibition of Aurora B activity may prolong the S phase in *Fzr1$^{-/-}$* cells. Moreover, EdU positive cells do not reflect a replication efficiency. Therefore, we will use a DNA combing assay to measure the replication speed in *Fzr1$^{-/-}$* and WT cells with or without Barasertib using IdU/ClU pulses.**

9. To test if Aurora B is active in Fzr -/- interphase cells, the authors perform IF for H3S10ph. First, interphase is not just G1 as suggested on page 8, but refers to G1/S/G2 (all phases except M phase). Second, it is surprising that interphase nuclear foci of Aurora B and H3S10ph were not observed in the Fzr+/+ MEFs (page 8). In many cell types Aurora B and H3S10ph foci become visible in late S phase and G2 (see for example: Ruppert et al, EMBO J (2018)). The authors should quantify the number of Aurora B/H3S10ph positive nuclei within the population of Cyclin A+ and Cyclin A- cells. It is expected that in the Fzr -/- MEFs this fraction is specifically increased in the Cyclin A-, and less so in the Cyclin A+ cells.

© 2020. Published by The Company of Biologists under the terms of the Creative Commons Attribution License (https://creativecommons.org/licenses/by/4.0/).

- **As suggested by Reviewer #3 (Minor point #6), IF images (Figure S7) were moved to Figure 5B-D.**
- **As pointed out by this Reviewer, interphase is not just G1 and all phases except M phase. Moreover, we did not precisely describe the results of IF. In response, we changed the sentence in "Results" to: "To asses CPC activity throughout the cell cycle, we examined the expression of H3 p-S10 in G1 where H3 p-S10 expression is not normally observed. A large number of interphase nuclear foci of H3 p-S10 and Aurora-B were observed in *Fzr1*$^{-/-}$ MEFs, compared to *Fzr1*$^{+/+}$ MEFs (Fig. 5B). It has recently been shown that nuclear foci of H3 p- S10 become visible in late S phase and G2 (Ruppert et al., EMBO J, 2018). Indeed, in *Fzr1*$^{+/+}$ MEFs with Cyclin A expression, nuclear foci of H3 p-S10 were observed in G2 cells (Fig. 5C). In *Fzr1*$^{-/-}$ MEFs, nuclear foci of H3 p-S10 were observed in G1 cells without Cyclin A expression (Fig. 5C)."**

In addition, as suggested by this Reviewer, we will quantify the number of Aurora- B/H3S10ph positive nuclei within the population of Cyclin A+ and Cyclin A- cells.

10. The high interphase levels of CPC proteins in the teratoma cell lines is interesting, but some functional consequence of having these high CPC levels in interphase (that can be (partially) reversed by ABK kinase inhibition) should be shown. Moreover, the authors should do a better job in showing that these high levels are indeed a consequence of Emi overexpression and thus APC/C cdh1 inhibition and increased protein stability. They should knock-down Emi1 (FigS8) and repeat the TB+RO release shown in 6A. Are the levels of Borealin and Aurora B now going down similar as in somatic (Hela) cells? And what happens to INCENP levels which is not a target of APC/C cdh1 according to figure 2D? In addition, as shown in figure S8C, mRNA levels of CPC members also appear to be high in these pluripotent stem cells. To assess the contribution of transcription/translation and protein stability to the high interphase CPC protein levels in these cells, the authors should test to what extent cycloheximide treatment affects protein levels of CPC proteins in these cells.

- **As suggested by Reviewer #3, we removed the last experiments with embryonal carcinoma cells (Figure 6 and Supplementary Figure S8). The comments from this Reviewer about the experiments using embryonal carcinoma cells will be very useful to complete follow-up studies.**

11. In figure 6D the authors show IF images of endogenous Aurora B, INCENP and Survivin in NCC- IT-A3 interphase cells. It is unclear why Flag-Borealin is overexpressed in these cells and if this Borealin overexpression could somehow affect the levels of the other CPC members. It is important to perform IFs for endogenous Aurora B, INCENP, Survivin (and preferentially endogenous Borealin) without exogenously expressed flag-Borealin. The levels should be compared to the levels in interphase HeLa cells by mixing the two cell lines. One of the two cell lines can be labelled through stable expression of fluorescently tagged H2B. By performing IFs for CPC proteins and Cyclin A on co-cultured HeLa and NCC-IT-A3 cells, the CPC protein levels in cyclin A+ (HeLa vs NCC-IT-A3) vs cyclin A-(HeLa vs NCC-IT-A3) cells can be quantified by quantitative IF.

- **Same response as to Major point #10**

12. In general, the discussion section is not very clear or logical. A few examples:
page 10/11: "Previous reports show that.....additional binding affinity for the APC/C. Therefore, Borealin may require residues W70 and W74 that are essential for the interaction with Survivin for additional binding affinity to Cdh1. However, Survivin does not act as an adaptor of Borealin ubiquitylation". >>> A conclusion or model explaining their findings would be helpful here.

- **As pointed out by this Reviewer, Borealin recognition by APC/C^{Cdh1} is complex. As shown in above (Major point #3), Borealin 5E+W70E/F74E can suppress ubiquitylation, whereas 3E, 3E+W70E/F74E (previous 5E), and 5E mutants do not. Borealin ubiquitylation may depend on its binding to Survivin as described above. We made a schematic model (please see Attached Figure 9). After completing data acquisition, we will modify this model and will add in Supplementary Figure.**

page 11/12: "Surprisingly, the forming the CPC for Aurora B activity is not always necessary. Upon mitotic exit, APC/C cdh1-mediated degradation of Borealin and/or Aurora B might

© 2020. Published by The Company of Biologists under the terms of the Creative Commons Attribution License (https://creativecommons.org/licenses/by/4.0/).

trigger the termination of CPC function and Aurora B activity. Indeed, we demonstrated that the Aurora B and Borealin could form CPC complex in interphase of EC cells with low activity of APC/C cdh1". I am unable to grasp the point/ rationale of this part of the discussion.

- **Same response as to Major point #10.**

Finally, the part on CPC overexpression, carcinogenesis and the role of Cdh1 in this (page 12) is a bit overstated and not supported by any of the data presented in the manuscript

- **As pointed out by this Reviewer, in our discussion of CPC overexpression and carcinogenesis, the role of Cdh1 was overstated and not supported by our data. Therefore, we will modify the sentence after checking the phenotype of replication and cell cycle of *FZR1*$^{-/-}$ MEFs expressing the Borealin non-degradable mutant (Major point #5).**

<u>Minor points:</u>

1. *Figure 1: The authors have indicated timepoint 12hrs as "S phase", it is unclear on what marker this is based. In other words, how do the authors know this is indeed the moment these Hela cells enter S phase?*

- **At 12 hrs after nocodazole release, the expression level of p27 decreased. Therefore, cells at this timepoint are in early S phase. To show the expression of Borealin and Aurora-B from S phase to G2/M, we provided a new experiment using double thymidine block release to show the expression in S phase in Figure 1A (please see Attached Figure 10).**

2. *Figure 2A (right panel): the lanes of Myc-Cullin do not match the rest of the blot (8 lanes in Myc- Cullin IP, whereas there are 7 lanes in the other rows). In addition, Myc-CUL2 was not expressed, and hence it cannot be excluded that CUL2 is involved in the degradation of borealin. This WB needs to be repeated.*

- **As pointed out by this Reviewer, the expression level of CUL2 was very low. Therefore, we will repeat this experiment to confirm that CUL2 is not involved in the degradation of Borealin.**

3. *It would be better to show the effects of Cdh1 overexpression on endogenous Borealin levels instead of overexpressed GFP-Borealin as shown in figure 2C. This is actually shown in U2OS with Dox-inducible cdh1 in figure S1B, this figure can be part of the main figure.*

- **As suggested, we replaced Figure S1B and Figure 2C.**

4. *Figure 2E: A control for functionality of the cdc20 knock-down is lacking (for instance lack of Ub of cyclin B). Moreover, there is still significant ubiquitination of flag-Borealin in the siCdh1 condition. Is this due to poor knock-down of Cdh1 or does cdc20 contribute? Would co-depletion of cdh1 and cdc20 further reduce the ubiquitination of Flag-Borealin?*

- **As described in the Minor comment #5 of Reviewer #2, we could not observe an increase in the level of Borealin in Cdh1 depleted cells. In this experiment, we also used cell lysates from asynchronized cells. Therefore, we will not see the increased level of substrates. To evaluate the functionality of the Cdc20 knock-down, we will examine the expression of Cyclin B in nocodazole release of Cdc20 depleted cells.**
- **As shown in Figure 2A, Cdc20 cannot bind Borealin. Moreover, we confirmed that Cdc20 overexpression has no effect on protein level of Borealin (Supplementary Figure S1B). Therefore, we believe that Cdc20 does not contribute to Borealin ubiquitylation.**

5. *Figure 3E and 3F are redundant, and 3F is more convincing than 3E as it shows Flag-Borealin 5E stabilization is not affected by translation rate. Figure 3E may be shown as a supplemental figure.*

- **As suggested, we moved from Figure 3E to Supplementary Figure S5B.**

6. *Materials and methods:*
It would help to mention from what kind of tissues PA1, MRC5 and NCC-IT-A3 were originally derived.

- **As suggested by Reviewer #3, we removed the last experiments with embryonal carcinoma cells (Figure 6 and Supplementary Figure S8).**

Where do all the different plasmids encoding Borealin come from? Please include references.

- **We provided the following reference showing the origin of plasmids encoding Borealin in “Materials and methods”.**

© 2020. Published by The Company of Biologists under the terms of the Creative Commons Attribution License (https://creativecommons.org/licenses/by/4.0/).

Gassmann R, Carvalho A, Henzing AJ, Ruchaud S, Hudson DF, Honda R, Nigg EA, Gerloff DL, Earnshaw WC (2004) Borealin: a novel chromosomal passenger required for stability of the bipolar mitotic spindle. *J Cell Biol* 166:179-191.

The authors should mention whether the Fzr-/- MEFS were derived from conditional or constitutive FZR knock-out mice.

- ***Fzr1-/-*** **MEFs from conditional knockout mice with a targeted mutation in the Fzr1 locus were provided by Dr. Marcos Malumbres (Spanish National Cancer Research Centre). We added this information in "Materials and methods".**

Reviewer #2

This Reviewer also believes that our findings are important and solid. She/he recognizes that "*The data on Borealin degradation by Cdh1 is robust and thorough, with a strong combination of in vivo and in vitro experiments.*", yet, he/she asks that we should address the following specific issues.

Major points:

1. *I have a number of issues with the interpretation of the S phase entry experiment shown in Figure 5B. Firstly, the figure legend does not contain any information on how many times the experiment was conducted and what the error bars represent.*

- **We added in "Figure legends" the information on how many times each experiment was conducted and what the error bars represent.**

Second, what is the rationale behind the peak value measuring the "efficiency of DNA replication"? Surely, this is not a measure of the total number of cells that complete S phase, as some of the cells are likely to have already completed S phase at the peak point and the actual peak may be missed if it is in between time points. Third, the effect of Barasertib on the FZR1- deleted cells appears to be a delay in S-phase completion, not a decrease in the number of cells that initiate replication early as claimed by the authors (the 3-21 hour time points are unchanged). With these three issues, the claim that "abnormal DNA replication in Fzr-/- MEFs was rescued by Aurora-B kinase inhibitor treatment" in the discussion is unfounded.

- **To solve these points, we will use a DNA combing assay to measure the replication speed in *Fzr1$^{-/-}$* and WT cells with or without Barasertib using IdU/ClU pulses as also suggested by Reviewer #1 (Major point #8). We will also change the discussion section after obtaining these results.**

2. *The microscopy in the manuscript requires quantification (Supplementary Figure 7 A-C ~~and Figure 6D~~). Single anecdotal examples are not enough to inform the reviewer of how robust the results are. The percentage of cells with H3S10 foci should be measured in each experiment, preferably with 3 biological replicates and statistical significance.*

- **As suggested by Reviewer #3, we removed the last experiments with embryonal carcinoma cells (Figure 6). As for Figure 5B-D (~~Supplementary Figure 7A-C~~), we will measure the percentage of cells with H3S10 foci in each experiment, preferably with 3 biological replicates and statistical significance (similar comment of Reviewer #1 Major point #9).**

3. *With the Borealin 5E mutant, the protein levels are greatly increased even at cell cycle stages where Cdh1 should not be active (t = 0 in Figure 3E and Figure 4A). Furthermore, the levels decrease after G1 entry to a similar extent to the wildtype Borealin. These data suggest that the increase in expression of the 5E mutant is not due to preventing Cdh1-targeted APC/C activity.*

- The expression level of the 5E mutant was always higher than that of wild type despite **transfecting the same amount of plasmid, suggesting that suppression of degradation may cause protein accumulation for the 5E mutant. As described in the Major points #3 of Reviewer #1, now we focus instead on the newly generated 5E+W70E/F74E mutant. We will repeat these experiments (Supplementary Figure S5B (previous Figure 3E) and Figure 4A) by using Borealin this new mutant. Moreover, we will compare Borealin stability by using a vector (please see right figure) that co-expresses both WT and mutant Borealin.**

© 2020. Published by The Company of Biologists under the terms of the Creative Commons Attribution License (https://creativecommons.org/licenses/by/4.0/).

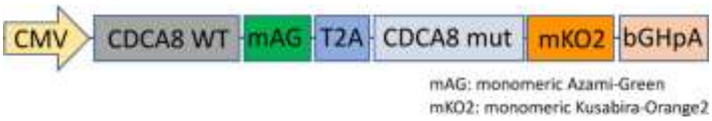

Minor comments:

1. *In Figure 2A, there appears to be an extra lane in the Myc-Cullin IP blot. It looks like there are 6 lanes in line with the other blots that have 5 lanes in the same width of blot.*

- Thank you for pointing out of our mistake. In this experiment, CUL2 expression was very low. Therefore, we will repeat this experiment.

2. *It would strengthen the argument of a direct interaction between Borealin and Cdh1 if the pull- down with reconstituted protein were repeated with the 5E mutant.*

- We will examine the pull-down assay using the new Borealin 5E+W70E/F74E mutant.

3. *Near the bottom of page 4, the authors claim "Borealin levels are low in G1 and rise during late S, G2, and M." However, the data cited for this (Figure 1A) only shows the M to G1 transition.*

- **We added the data from a double thymidine block release to show the M to G1 transition (same comment from Minor point #1 of Reviewer #1).**

4. ***In Figure 1C, why do the authors use two general proteosome inhibitors, but not the more specific APC/C inhibitor proTAME?***

- **As recommended by this Reviewer, we will use the specific APC/C inhibitor, proTAME, and evaluate its effect on Borealin levels in G1.**

5. *In Figure 2E, when the ubiquitination is decreased with Cdh1 depletion, why are the overall levels FLAG-Borealin not increased? Is this a result of overexpression?*

- **In Figure 2E, we used cell lysates from asynchronous cells. Therefore, we could not observe an increase in the level of Borealin in Cdh1-depleted cells. As shown in Figure 2D, increased Borealin levels were observed in Cdh1 depleted G1 cells.**

6. *The rational for the design of the experiment shown in Figure 3F is not explained in the text.*

- **As the pointed out by this Reviewer, we added a better explanation of Figure 3E (previous Figure 3F) in the "Results" section. We now state: "Moreover, the half-life of the 5E mutant was longer than that of wild type in G1". However, we will repeat this experiment using the Borealin 5E+W70E/F74E.**

7. *In supplementary figure S8C, it is not clear that the Aurora B levels decrease after Emi1 inhibition as claimed in the text.*

- **As suggested by the Reviewer #3, we removed the last experiments with embryonal carcinoma cells (Figure 6 and Figure S8).**

8. *The data in Figure 2D shows that INCENP levels decrease in G1 even when Cdh1 is inhibited. How do the authors explain the Aurora B activity observed in G1 upon FZR1 deletion when the essential kinase activator is still missing at this stage?*

- Reviewer #1 also raised this point (Major point #1). Please read my comments described in Major point #1 of Reviewer #1.

Reviewer #3

This Reviewer stated that "*Overall, the study is well conducted, and the data shown largely support the conclusions drawn in the text.*" He/she asks that we address the following specific issues.

Major points:

1. *It is not quite clear why the authors decide to study the consequences of not having Cdh1 in Fzr1-/- MEFs instead of performing RNAi in human cells. They come to the conclusion that cells lacking Cdh1 enter S-phase prematurely (as previously described), and that this is due to the presence of CPC activity too early in the cell cycle. This conclusion is supported by the fact that the premature entry into S-phase can be partially alleviated by treating the cells with the Aurora B inhibitor Barasertib. However, this experiment does not provide any direct evidence that the CPC is directly involved in the regulation of S-phase. Loss of Cdh1 is expected to derail many*

© 2020. Published by The Company of Biologists under the terms of the Creative Commons Attribution License (https://creativecommons.org/licenses/by/4.0/).

aspects of cell cycle control, and the fact that inhibition of Aurora B, another mitotic enzyme with many targets, alleviates the loss of Cdh1, does not necessarily mean that Aurora B directly promotes early S-phase, and to my knowledge, there is no evidence that the CPC in any way regulates S-phase entry. A better experiment would be to use human HeLa Flp-In cells to conduct an RNAi rescue experiment and replace the endogenous Borealin with the Borealin mutant that cannot be ubiquitinated and then ask what the functional consequences are of this situation. If again, a premature entry into S-phase is observed, it could then be checked how this affects the rest of the cell cycle. Do you get DNA-bridges, indicative of incomplete replication?

- **This comment is very similar to that from Reviewer #1 (Major point #5). We will examine mitotic and cell cycle defects after replacement of endogenous Borealin by the new Borealin 5E+W70E/F74E mutant (siRNA knock-down and add-back experiment). In this experiment, we will carefully check DNA-bridges.**

The last experiment in the manuscript seems like an unnecessary add-on, the experiments in the pluripotent stem cells do not come to any clear conclusions and would better be left out.

- As suggested, we removed the last experiments with embryonal carcinoma cells (Figure 6 and Supplementary Figure S8).

Minor points:

1. *The title: The title should be changed. The authors do not provide any clear evidence that CPC activity is maintained when the CPC is not degraded.*

- This Reviewer suggests that we change the title. After obtaining all the data, we will change the title.

2. *Figure 2: the Myc-Cullin 2 expression is very poor.*

- **Reviewer #1 also raised this point (Minor point #2). We will repeat this experiment.**

3. *Figure 3: The rationale for choosing the residues for mutational analysis is not clearly explained.*

- **Reviewer #1 also raised this point (Minor point #12). To help explain our reasoning, we provided a schematic model for explaining our findings (please see Attached Figure 9). After completing data acquisition, we will modify this model and will add in Supplementary Figure.**

4. *Figure 3E: Since the mutated Borealin is expressed at a higher level, should the ratio of Flag- Borealin/actin not be higher for the 5E mutant? Or have both WT and 5E ratios at 0h of Noc release been normalised to 1?*

- **Reviewer #2 also raised this point (Major point #3). The expression level of the 5E mutant was always higher than that of wild type despite transfecting the same amount of plasmid, suggesting that suppression of degradation may cause protein accumulation of the 5E mutant. Now we will focus on the new 5E+W70E/F74E mutant (please see my comments to the Major points #3 of Reviewer #1). We will repeat this experiment by using a vector that co-expresses both Borealin WT and 5E+W70E/F74E.**

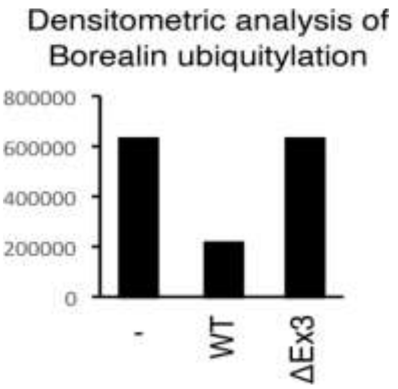

5. *Figure 4B: The result in Figure 4B should be quantitated.*

- **As suggested, we quantitated the ubiquitylated bands as shown in the right graph. We added this graph to Figure 4B.**

© 2020. Published by The Company of Biologists under the terms of the Creative Commons Attribution License (https://creativecommons.org/licenses/by/4.0/).

6. *Figure 5A: Can the authors provide a nicer blot for survivin? IF images would be really useful to back up the claim that the CPC is not degraded in the absence of Cdh1/Fzr1. The IF images from S7 could be put into figure 5.*

- **As suggested, we will repeat this experiment to show a better blot for Survivin in Figure 5A. Moreover, as suggested by the Reviewer, the IF images (Figure S7) were moved to Figure 5B-D.**

7. *Figure 5B: Could the authors show representative images in addition to the graph in Figure 5B? What about subsequent changes to cell cycle progression, e.g. compromised chromosome segregation, e.g. DNA bridges?*

- **In Figure 5B, we examined the number of EdU positive cells by flowcytometry. Therefore, we could not provide representative images. However, as suggested by the Reviewer #1 (Major point #8) and the Reviewer #2 (Major point #1), we will measure the replication speed in Fzr -/- and WT cells with or without Barasertib using IdU/ClU pulses and investigate changes in cell cycle progression. To see subsequent changes to cell cycle progression, such as compromised chromosome segregation or DNA bridges, we will carefully evaluate these phenotypes in *FZR1*$^{-/-}$ cells by immunofluorescence analysis.**

8. *Figure 6D: Why do the authors look at overexpressed Flag-Borealin instead of the endogenous Borealin?*

- **As suggested by this Reviewer, we removed the last experiments using embryonal carcinoma cells (Figure 6).**

Attached Figure 1

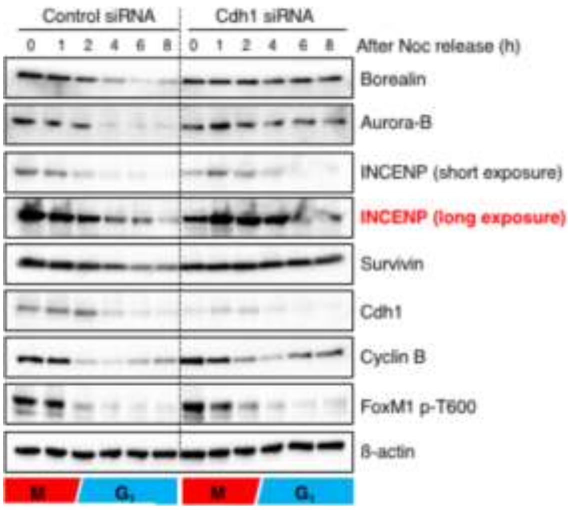

Attached Figure 2

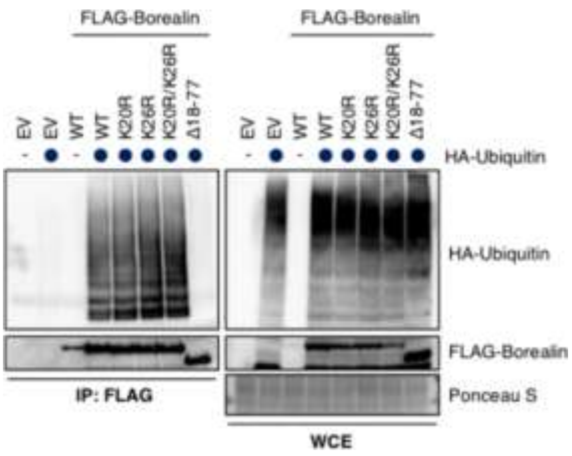

© 2020. Published by The Company of Biologists under the terms of the Creative Commons Attribution License (https://creativecommons.org/licenses/by/4.0/).

Attached Figure 3

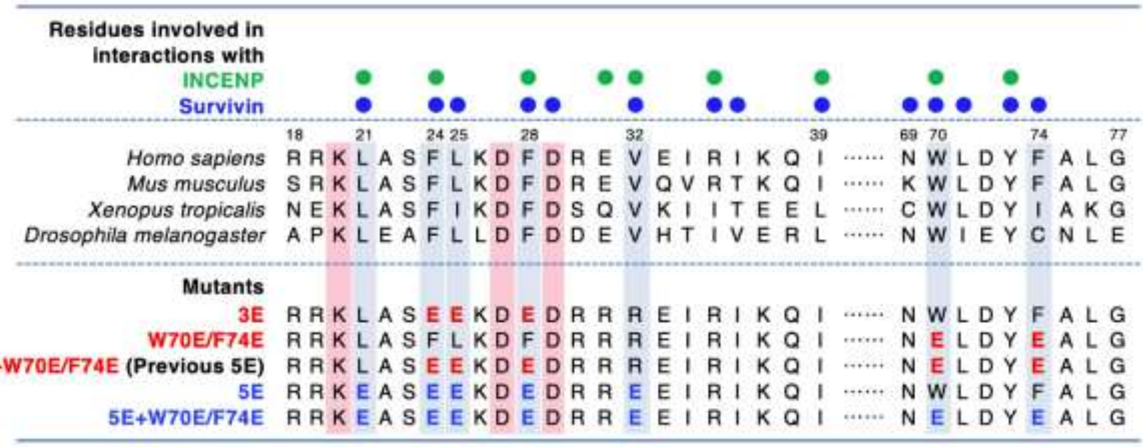

Figure provided for reviewer has been removed. It showed Fig. 3A from Jeyaprakash et al. (2007) Structure of a Survivin-Borealin-INCENP Core Complex Reveals How Chromosomal Passengers Travel Together. Cell 131, 271-285. (doi:10.1016/j.cell.2007.07.045)

The alignments include orthologs from *Homo sapiens, Mus musculus, Xenopus tropicalis, Drosophila melanogaster*. Conserved hydrophobic residues in Borealin are highlighted in blue and other conserved residues in Borealin are highlighted in pink. Above the sequences, coloured circles identify residues involved in interactions with Survivin (blue) and with INCENP (green).

Attached Figure 4

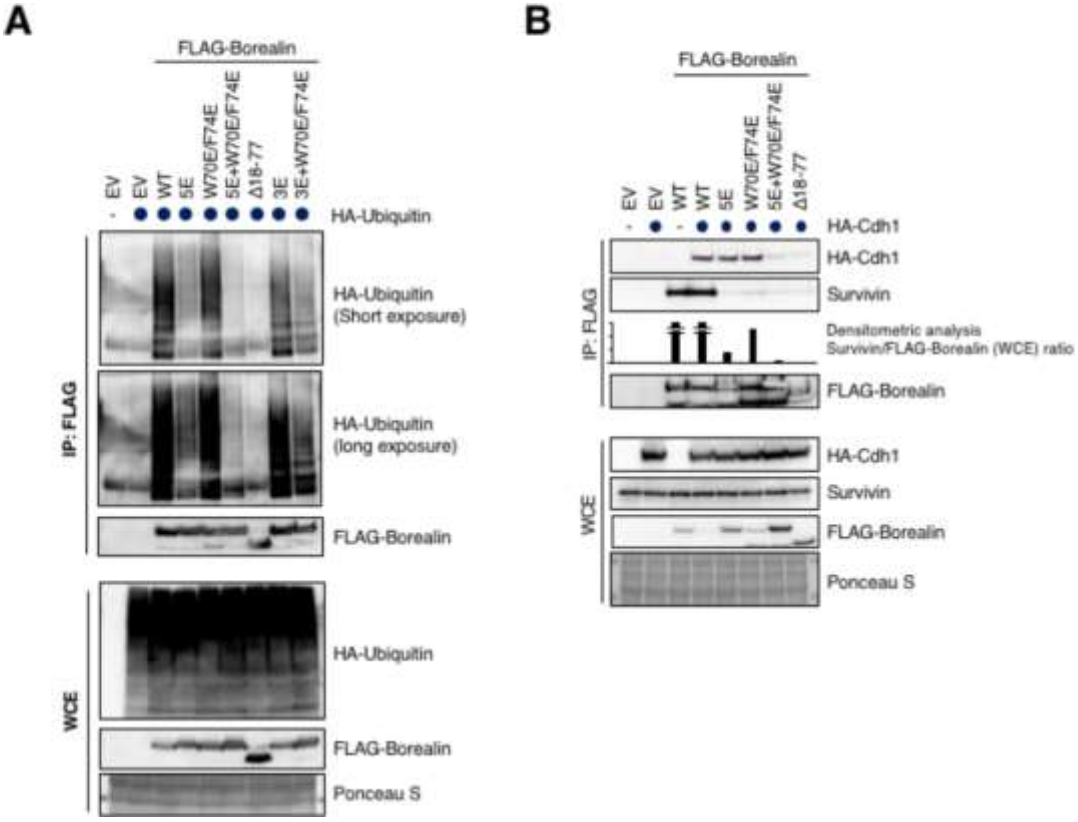

© 2020. Published by The Company of Biologists under the terms of the Creative Commons Attribution License (https://creativecommons.org/licenses/by/4.0/).

Attached Figure 5

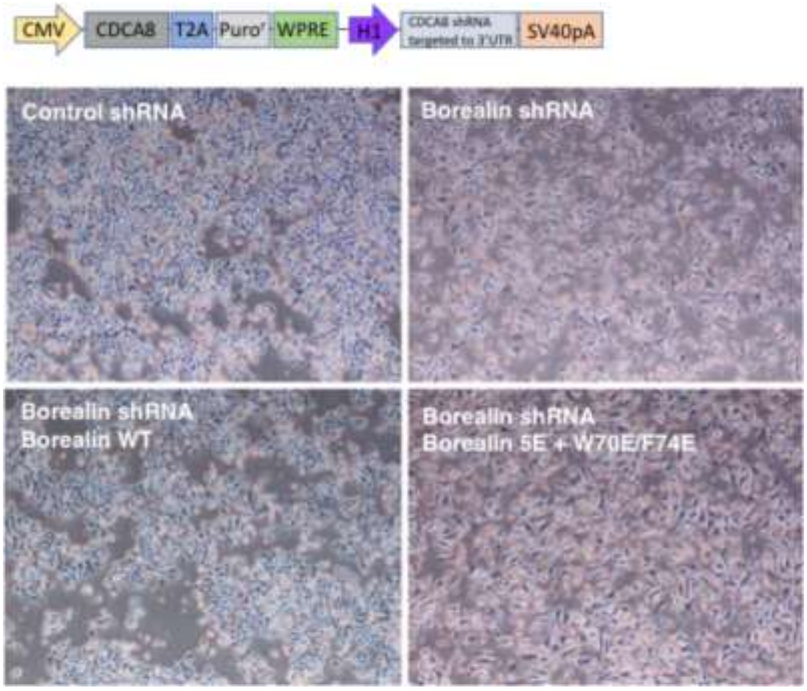

Attached Figure 6

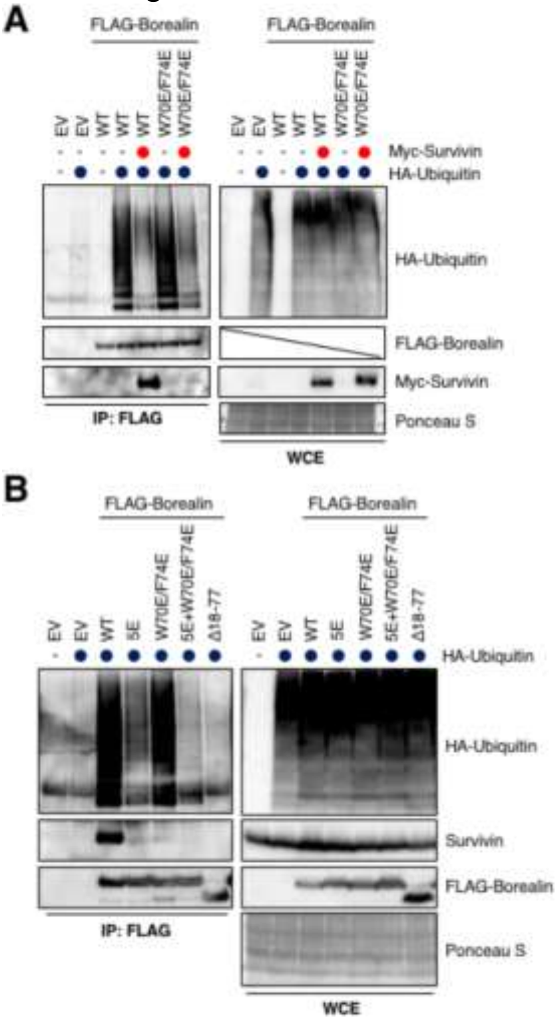

© 2020. Published by The Company of Biologists under the terms of the Creative Commons Attribution License (https://creativecommons.org/licenses/by/4.0/).

Attached Figure 7

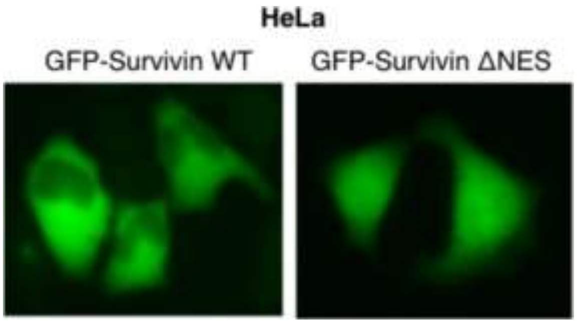

Attached Figure 8

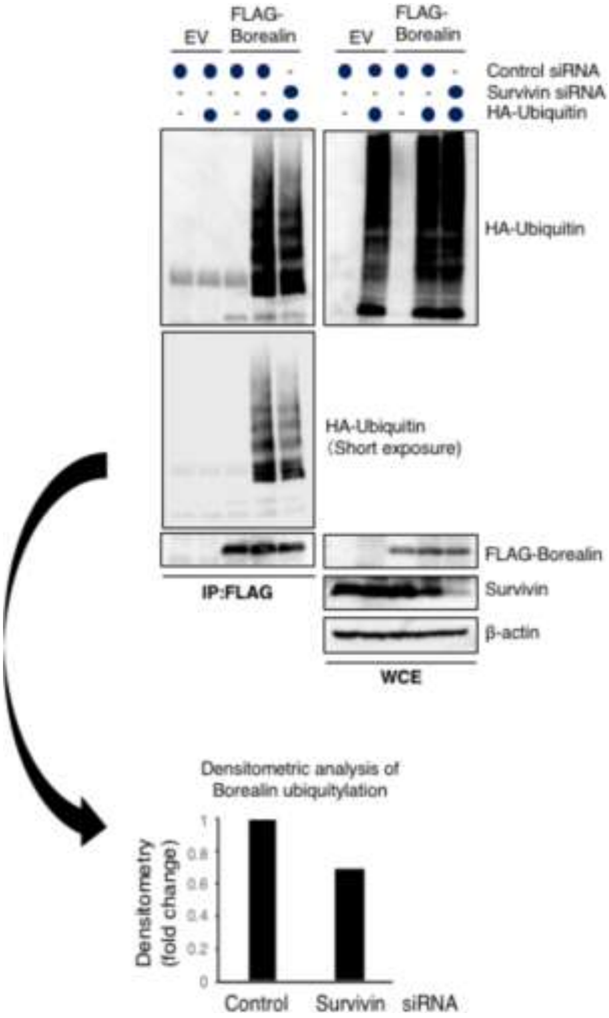

© 2020. Published by The Company of Biologists under the terms of the Creative Commons Attribution License (https://creativecommons.org/licenses/by/4.0/).

Attached Figure 9

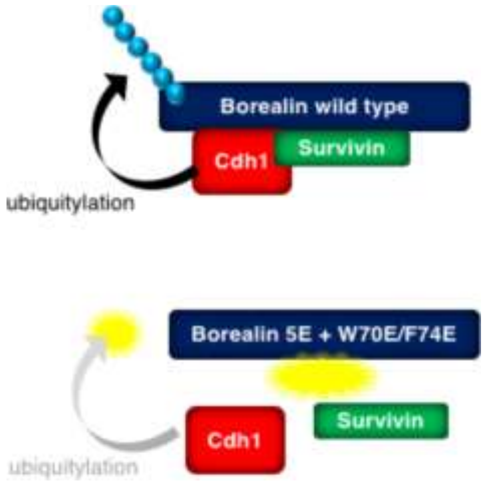

Attached Figure 10

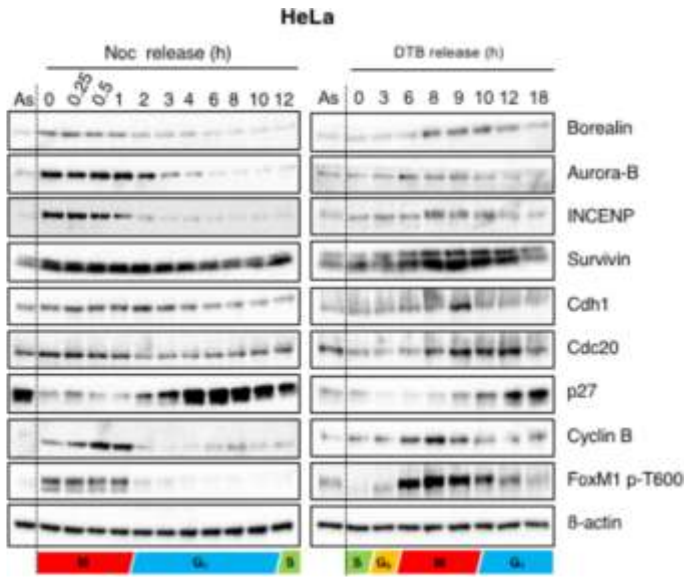

First decision letter

MS ID#: JOCES/2020/251314

MS TITLE: APC/C-Cdh1 is required for the termination of CPC activity upon mitotic exit

AUTHORS: Yasusei Kudo, Takaaki Tsunematsu, Rieko Arakaki, Hidehiko Kawai, Jan Ruppert, Koichi Tsuneyama, Naozumi Ishimaru, William C Earnshaw, and Michele Pagano
ARTICLE TYPE: Research Article

Thank you for sending your manuscript to Journal of Cell Science through Review Commons. I am happy to tell you that your manuscript has been accepted for publication in Journal of Cell Science, pending standard ethics checks.

© 2020. Published by The Company of Biologists under the terms of the Creative Commons Attribution License (https://creativecommons.org/licenses/by/4.0/).
